# Thermoresponsive Chitosan-Grafted-Poly(*N*-vinylcaprolactam) Microgels via Ionotropic Gelation for Oncological Applications

**DOI:** 10.3390/pharmaceutics13101654

**Published:** 2021-10-11

**Authors:** Lorenzo Marsili, Michele Dal Bo, Federico Berti, Giuseppe Toffoli

**Affiliations:** 1 Department of Chemical and Pharmaceutical Sciences, University of Trieste, Via Licio Giorgieri 1, 34127 Trieste, Italy; fberti@units.it; 2 Experimental and Clinical Pharmacology Unit, CRO National Cancer Institute IRCCS, Via Franco Gallini 2, 33081 Aviano, Italy; mdalbo@cro.it (M.D.B.); gtoffoli@cro.it (G.T.)

**Keywords:** microgels, thermoresponsivity, drug delivery, smart delivery systems, biodegradable polymers

## Abstract

Microgels can be considered soft, porous and deformable particles with an internal gel structure swollen by a solvent and an average size between 100 and 1000 nm. Due to their biocompatibility, colloidal stability, their unique dynamicity and the permeability of their architecture, they are emerging as important candidates for drug delivery systems, sensing and biocatalysis. In clinical applications, the research on responsive microgels is aimed at the development of “smart” delivery systems that undergo a critical change in conformation and size in reaction to a change in environmental conditions (temperature, magnetic fields, pH, concentration gradient). Recent achievements in biodegradable polymer fabrication have resulted in new appealing strategies, including the combination of synthetic and natural-origin polymers with inorganic nanoparticles, as well as the possibility of controlling drug release remotely. In this review, we provide a literature review on the use of dual and multi-responsive chitosan-grafted-poly-(*N*-vinylcaprolactam) (CP) microgels in drug delivery and oncological applications.

## 1. Introduction

One of the main efforts in biomedical research focuses on the design of new nano- and microstructured, biocompatible materials not necessarily from natural origin. In this field, gels, microgels and nanogels have become popular due to their high biocompatibility and the significant progress in the field of polymer synthesis [1,2]. Gels are defined as three-dimensional polymeric networks swollen by the presence of a solvent [3]. The type of crosslinking can be of physical or chemical origin, depending on the type of the interaction between different chains. If water is the dispersion medium, a gel is commonly referred to as a hydrogel [3]. Over the years, cross-linked polymers have been prepared in a wide range of compositions, structures and typologies. Furthermore, numerous efforts have focused on the development of finely dispersed cross-linked polymeric particles with a size range from a few nm to several μm.

Polymer particles and hydrogels have been extensively studied as they can provide biocompatible scaffolds for the application of nanoparticles [4,5,6,7]. Polymeric systems have also been investigated for the possibility of creating “smart” drug delivery systems [8,9,10,11] capable of responding to external stimuli such as variations in pH [4,11,12,13,14,15], temperature [1,11,12,16,17,18,19,20,21,22,23,24,25], magnetic fields [19,26,27,28,29,30,31,32] and concentration [33,34,35]. Some of these systems have proved to be particularly promising due to the possibility of providing drug and nanoparticle encapsulation, along with the sensitivity for an external trigger [20,21,27,36]. Their biological interest consists of the presence of a desirable sharp transition. This allows the control of the conformational and dimensional properties of the delivery devices according to the structural characteristics of the polymer and the solution environment. This type of device has opened a new generation of anti-cancer drug delivery systems that exploit the more acidic and hypoxic microenvironment of solid tumours [37,38,39,40], as well as their anormal vascular morphology, to achieve better targeting and treatment efficacy. The incorporation of nanoparticles with super-paramagnetic properties (MNPs) or gold nanoparticles (Au NPs) has provided the basis for dual- or cascade-triggered therapies [20,27,36,41]. Dual pH/thermo-responsive stimuli-triggered delivery systems have been extensively reported [42,43,44,45] as theranostic agent for tumour treatment and localisation [18,46,47]. Block copolymer micelles stimuli-responsive systems have been employed for the delivery of a broad range of therapeutics through effective sensing of targets in the body [48,49,50]. Several micelle formulations were demonstrated to be clinically effective and allowed the modulation of the activity of encapsulated drugs at the subcellular level [51]. Nonetheless, a better understanding on the behaviour of these systems is crucial in order to adapt them for the utilisation in conventional cancer therapies.

In oncological research, nanoparticles-based therapies have been developed for the treatment of carcinomas through a variety of methods of administration [9,29,52,53,54,55,56,57,58,59,60]. The interest towards nano-based therapies in oncological research is to develop minimally invasive treatments that can enhance selectivity towards tumour tissues. The utilisation of metal and polymeric particles with well-defined size and shape may improve the process of drug delivery, resulting in the reduction of the strong side effects associated with the utilisation of chemotherapy drugs [61,62,63,64,65,66,67,68,69,70,71,72,73,74,75]. Current research on nanoparticles for cancer diagnosis and application has established its advantages within contemporary oncology, as well as its intrinsic limitations [52,76,77,78]. Consequently, notwithstanding the astonishing technological progress made in the field of nanomedicine, standard oncological treatments still rely on conventional methods, such as surgery, radiotherapy and chemotherapy [64,79,80,81,82,83,84,85]. To this date, clinical application of nanoparticles is still hindered by the lack of understanding of the mechanism of interaction between nanoparticles and the complex biological matrix and by the lack of trials and standardisations [9,52,54,56,57,80,86,87,88,89,90,91,92,93,94,95].

In the present review, we provide a general definition of polymeric nano- and microgels. Several applications of polymeric nano/microgels in the biomedical field are described, including those utilising chitosan (CS) and thermoresponsive polymers such as poly(*N*-vinylcaprolactam) (PNVCL) and poly(*N*-isopropylacrylamide) (PNIPAM). A detailed focus regarding the preparation of thermoresponsive chitosan-g-PNVCL (CP) based nano- and microgels as drug delivery systems is also provided.

## 2. Microgels: Polymeric Nanoparticles or Microparticles?

**Figure 1:**
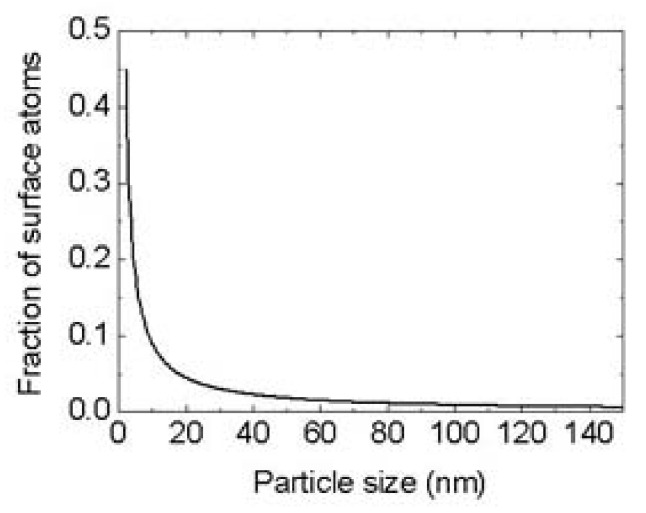
Exponential decay of surface atoms as a function of particle size for cubic shape (α = 6) and interatomic distance a = 1.5 Å. Reproduced from [98], Hindawi 2015.

According to the IUPAC definition, a microgel is a “*particle of gel of any shape with an equivalent diameter of approximately 0.1 to 100 μm*”. This definition is recommended as it introduces the critical size of 100 nm in order to distinguish between a nanogel and a microgel [3]. The same guidelines are provided for the distinction between “nanoparticles” and “microparticles” [96]. The utilisation of the prefix “micro” provides a clear indication that the material possesses a very low percentage of surface atoms (Figure 1). Consequently, surface properties of microgels are closer to bulk material than to nanomaterials that are a few nm in size [97,98,99,100]. Conversely, nanoparticles that are a few nm in size have superficial properties similar to those of polymer chains, being in the same dimensional range.

However, the term “nanogels” have been extensively used for the description of gel particles with a size inferior to 1000 nm [7]. This semantic problem may have been developed due to the growing interest in nanotechnologies and the phenomenon of “me too science” [101], which resulted in a general preference for nano-related publications. However, a remarkable amount of in vivo studies have outlined that nanoparticles are rapidly subjected to a metabolic fate depending on their size [102,103,104,105,106]. Most metal particles developed for theranostic purposes usually exhibit interesting properties in a dimensional range inferior to 10 nm, since surface properties arise from the exponential increase of the number of surface atoms that occurs under a critical size threshold [96,97,98,99]. However, nanoparticles in this dimensional range are rapidly disposed by the body through the kidneys [31,107,108,109,110,111]. Conversely, one of the main reasons for the development of gel particles with a size >100 nm is their low surface activity or, according to official recommendations, their being “micro” rather than “nano”. In fact, the majority of the colloidal systems used for clinical applications consist of formulations of particles between 100 and 1000 nm [9,29,52,53,54,55,56,57,58,59,60,76,77,78,107,112,113]. Although it is true that inorganic nanoparticles are used in some formulations, they are always enclosed within systems that easily fit within the scale of “micro” (>100 nm). The utilisation of microgels as an enclosure of inorganic nanoparticles improves their biostability, biodegradability and toxicity and prevents them from being easily removed from the body, thereby improving their therapeutic effectiveness [4,5,6,32,36,108,109,110,111,114].

The reduced surface activity of microgels makes them more biomimetic materials than nanoparticles, and their dimensions are close to the size range of cell vesicles [102,115,116] and proteins. This, together with the use of biocompatible and stimuli-responsive materials for their preparation, makes microgels ideal vehicles for the fabrication of drug delivery systems. Furthermore, their biomedical applications are bound to their metabolic fate, which is heavily influenced by their size [117,118,119,120,121]. The utilisation of microgels allows the avoidance of cytotoxic effects associated with “big” particles (>500 nm). At the same time, microgels are more suitable for in vivo application in relation to small particles (<50 nm) that are subjected to rapid renal excretion. Initially, the miniaturisation of delivery devices was focused on the development of micrometric particles (>1 μm) [103,104], which still represent the base of many commercial formulations and “innovative” therapies. For some oncological applications, such as chemoembolisation, this paradigm has not yet changed, and micrometric particles are used [105,106]. However, by further miniaturising medical devices, some limitations related to the utilisation of bigger particles could be overcome. One of these important limitations is the treatment of advanced tumours [105].

Long-lasting circulation is another important feature of microparticles and microgels with an overall size around 100 nm [122]. This increases the possibility of the microparticle extravasating through fenestration tissues present in tumours, which are usually between 380 and 780 nm in size [117]. This phenomenon, called “passive accumulation”, led to the development of the “enhanced permeation and retention” (EPR) effect [87,89,115,116,117,118,119,120]. Accordingly, particles that are smaller in size are able to selectively accumulate in tumour tissues and are retained due to poor lymphatic drainage, and their utilisation may improve cancer therapy. The EPR effect is often described as a controversial concept [75,76,121] due the small percentages of the total administered nanoparticles that are usually able to reach the tumour [123]. As a matter of fact, non-continuous epithelia are present also in the liver, with vascular fenestrations between 50 and 100 nm, and in the spleen, due to the presence of interendothelial cell slits between 200 and 500 nm [122]. For these reasons, some studies reported that particles between 70 and 200 nm have the ideal size to accumulate passively within tumour tissues [117,124,125]. A “golden dimension”, in fact, does not exist, and the ideal size threshold is often subject to interpretation. Probably, the reference to this “ideal size” was originated by a study published in 1990 study by Klibanov et al. that described the long circulation times of PEG-grafted liposomes [126]. However, although the advantages of the utilisation of liposomes in drug delivery have been extensively demonstrated, it cannot be excluded a priori that these size thresholds can change depending on materials and type of particle used. Also, the ability of nanoparticles to accumulate in target tissues can depend on the tumour type and is influenced by many micro-environmental factors. A remarkable amount of in vivo studies have outlined that nano and microparticles are rapidly subjected to a metabolic fate depending on their size [117,118,119,120,121] in the absence of an active targeting system that recognises a specific characteristic of the tumour [124,127,128,129], even though active targeting does not necessarily represent the best choice for tumour targeting [130].

In addition to oncology, the ability to produce smaller nano- and microgels has opened up new possibilities for the treatment of other pathologies (e.g., osteoarthritis [131], schizophrenia [132], bacterial infections [133], vaccine delivery [93,134]) and for cosmetic applications. Systems of such dimensions, such as liposomes, are similar in size to microgels and are usually prepared in a range between 50 and 250 nm. To date, they are present in many commercial formulations and are present in most drugs based on “nanotechnologies” that are widely used in oncological hospitals (e.g., Liposil [122]). Another recent example of these drugs is Pfizer’s SARS-CoV-2 vaccine, consisting of mRNA filaments encapsulated inside a liposomal envelope [135].

## 3. Thermoresponsive Microgels

### 3.1. Volume Phase Transition Temperature

**Figure 2:**
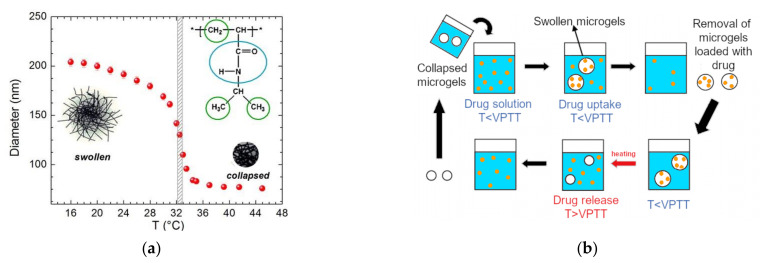
Heating cycles for poly(*N*-isopropylacrylamide)-based microgels (red). (**a**) Variation of hydrodynamic diameter with temperature; Adapted with permission from Corezzi [147], copyright by Elsevier, 2016. (**b**) Schematic representation of the temperature-induced drug-release process from poly(*N*-isopropylacrylamide) or poly(*N*-vinylcaprolactam) microgels.

In oncology, d-triggered drug release is of particular interest as it allows controlled and gradual drug release. In gels, volume phase transition (VPT) is characterised by an discontinuous and abrupt change in the degree of swelling that may arise from the coexistence of two gel phases that differ in the degree of swelling [136]. The application of an external trigger would cause the polymer meshes within the structure of the microgels to swell or shrink (Figure 2), thereby releasing the encapsulated drug. The transition temperature of thermoresponsive microgels is referred to as “volume phase transition temperature” (VPTT) [137].

However, the relation between the lower critical solution temperature (LCST) of the thermoresponsive polymer and the corresponding VPTT of the microgels represent a subtle problem in the characterisation of thermoresponsive microgels [22,137,138,139]. Both transition temperatures are determined by the balance of interactions between hydrophilic and hydrophobic segments [140]. As a matter of fact, it has been reported that hydrogels could be considered concentrated polymer solutions whose concentration could be calculated from the amount of water retained by the hydrogel network [138]. Nevertheless, the LCST is still widely used to refer to the transition temperature of microgels [12,14,16,18,20,30,141,142]. Theoretically, if we consider gels concentrated polymer solution, the VPTT coincides with the LCST if the LCST is not affected by polymer concentration (type II polymer, e.g., PNIPAM) [24,143,144,145]. The possibility of distinguishing LCST from VPTT allows us to understand the possible release mechanism of the microgels. It has been reported the average hydrodynamic diameter of microgels of a thermoresponsive polymer exhibiting LCST, such as PNVCL and PNIPAM, decreases as the temperature increases (Figure 2a) [139]. This is in line with the fact that, above the VPTT, the interaction between polymer chains and solvent becomes unfavorable, and the microgel structure collapses. The observation of the polymer behavior (LCST) would have led to the opposite result, as the lowering of solubility results in the formation of aggregates. Following this line of reasoning, thermoresponsive microgels that are formed with polymers that exhibit upper critical solution temperature (UCST) would exhibit swelling behavior at VPTT. Thermoinduced delivery processes are based on the exploitation of the different ability of microgels to bind or retain drugs depending on the temperature of the systems (Figure 2b). If the polymer exhibits LCST, collapsed microgels are placed in an aqueous solution below VPTT. Accordingly, this induces microgel swelling and allows the drug to penetrate the pores of the microgel network. In order to be able to retain the drug, it is fundamental that the polymer–drug interaction is favorable. A more detailed discussion of drug–polymer interaction will be provided in chapter 6. After the removal of drug-loaded microgels, the product can be stored or freeze-dried for subsequent application. Drug delivery test are usually performed below and above VPTT in order to compare different release profiles in different conditions. Above VPTT, microgels shrinking results in a sudden increase of the encapsulated drug [146].

### 3.2. Microgel Characterisation

In biology-related publications, microgels are usually characterised by using dynamic light scattering (DLS) equipment accessible within biological laboratories, and their response to temperature is characterised by a dramatic change in their size distribution. However, one major limitation of DLS is that it provides the measurement of a hypothetical hard sphere that diffuses with the same speed as the particle under examination [148]. From the IUPAC definition of microgels, it can be inferred that microgels can be spherical, but they can have different shapes. Even when considering a spherical microgel, the size distribution provides the average D_h_, which is different from the real diameter of microgels. In practice, polymeric microgels are non- or quasi-spherical particles with a big solvation shell. Therefore, the size calculated from their diffusional properties represents the apparent size of the solvated particle. In this condition, it is very difficult, if not impossible, to establish the actual shape of a nano- or a microparticle with the means available in a biological laboratory, especially if the particle is made of amorphous material. Nevertheless, the extensive use of DLS for the characterisation of microgels makes it possible to compare the hydrodynamic diameters of prepared particles with similar methods and conditions even if their shape has not been yet analysed.

Microgels’ shape can be estimated through computer simulations that consider the interactions taking place in a certain environment [149]. A widespread approach to observe the morphology of microgels is the use of microscopy techniques. The use of optical microscopy has been reported for the observation of microgels with >1 μm diameter at different temperatures [138]. The observation of smaller samples is widely documented by TEM and SEM. However, the use of these techniques is not suitable for the observation of samples in solution or to establish their response to a temperature change. For polysaccharide-based gels, it is known that sample preparation can lead to irreversible aggregation or to a substantial contraction of their diameter due to dehydration. Particle fusion has also been reported during TEM observation [150]. Cryo-TEM allows the direct investigation of the particle morphology in solution [151,152] even though the analysis of aqueous dispersions requires method development for different sample types due to the low temperature in which the sample is kept during observation.

Small-angle X-ray (SAXS) and neutron scattering (SANS) are regarded as very useful and adequate techniques for the study of the overall shape and structural transitions of macromolecules and particles in solution [152,153,154]. However, the quantitative characterisation of heterogeneous and polydisperse systems may represent a difficult task and requires complex data analysis. SAXS has been used for the analysis of microgels prepared with precipitation polymerisation, which generally exhibit low polydispersity [155]. SANS is highly regarded for providing information about microgels’ internal structure and monomer distribution [156]. However, these techniques are not usually considered in biology-related publications. This has eventually led to difficulties in standardising polymer particles due to the difficulty of controlling their shape. This is particularly evident for microgels that are prepared using polymers of natural origins through self- assembly reactions, such as ionotropic gelation [157,158]. To date, there is a growing interest in the application of SAXS and SANS techniques for the detailed study of self-assembled microgels of natural origins [159,160], even though quantitative characterisation can be difficult due to their inherent polydispersity [161].

### 3.3. Multi-Responsive Microgels

Over the years, microgels able to respond to different types of triggers (pH [162,163,164], temperatures [1,11,12,16,17,18,19,20,21,22,23,24,25,165], magnetic fields [19,26,27,28,29,30,31,32] and concentration gradients [33,34,35]) have been prepared in various sizes and compositions, depending on the application they were designed for.

Several pH-responsive microgels have been proposed in oncology. These biomaterials are expected to store the drugs in normal pH and release them in presence of the mildly acidic microenvironment surrounding the tumour [162,163,164]. This is generally achieved by introducing pH-sensitive materials into the three-dimensional gel structure, such as poly(metharylic acid) (PMAA) [162,166]. Thermoresponsive synthetic materials, such as PNVCL or PNIPAM, have been proposed to induce drug release through a local increase in temperature [1,11,12,16,17,18,19,20,21,22,23,24,25]. However, this requires the presence of a structural element, such as gold nanoparticles (Au NPs), within the gel structure to control the release remotely. Although synthetic polymers represent suitable materials for the fabrication of nano- and microgels, they show a lack of biodegradability, thus limiting their use for in vivo applications [167,168].

Recent achievements in the fabrication of biodegradable alternatives have provided new appealing strategies for the design of new polymeric delivery systems, such as the combination of synthetic polymers with biocompatible polymers of natural origins [169]. However, what could really make this type of technology a breakthrough in oncology is the ability to “drive” release and control it remotely. The development of dual-responsive microgels provided a step forward in this direction [20,27,36,41]. CS, usually described as a responsive pH material, has been combined with various polymers for the fabrication of multi-responsive microgels. By combining CS with a thermoresponsive material such as PNIPAM or PNVCL, pH- and thermoresponsive microgels can be fabricated [12,14,18,20,21,170]. Au NPs and superparamagnetic iron oxide nanoparticles (SPIONs) have also been encapsulated within double-responsive polymeric microgels to fabricate hybrid materials exhibiting multi-responsivity [18,20,171]. Au NPs provide local heating upon exposure to near-infrared (NIR) frequencies. As particles heat, the thermoresponsive network reacts, and the drug is released by the microgel core. In this way, it is possible to carry out a combinational therapy based on both drug release and thermal-induced apoptosis. On the other hand, magnetic microgels loaded with SPIONs can produce local heating upon exposure to an alternating magnetic field. This causes a relaxation or collapse of the gel chains, inducing the release of the drug contained within the gel.

## 4. Preparation of Microgels via Ionotropic Gelation

**Figure 3:**
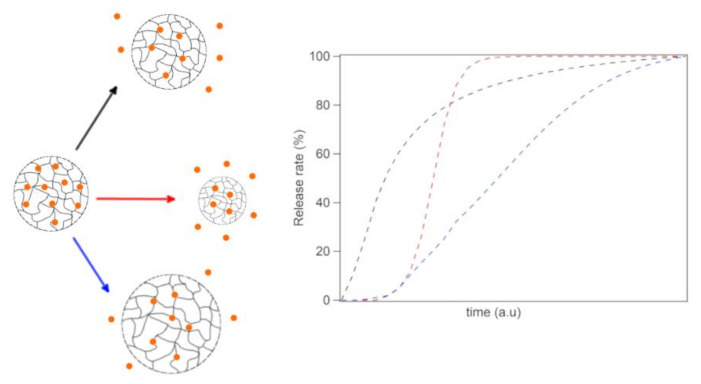
Representation of release mechanisms: diffusion (black), erosion (red) and swelling (blue) and related release curve according to different mathematical models.

Ionotropic gelation probably represents the most studied preparation for chitosan (CS)-based microparticles and nanoparticles (Figure 3). The process exploits the sol–gel transition of CS polymers in the presence of a polyanionic crosslinking agent, such as sodium tripolyphosphate (TPP). Due to the absence of toxic reagents, low energy requirements and the presence of mild and aqueous processing condition simplicity, the process has been extensively researched, becoming one of the standard encapsulation methods for the preparation of CS nanoparticles, in particular for drug delivery, gene therapy application and protein formulation [172]. The formation of micro- and nanoparticles through ionotropic gelation is based on the weak basic properties of the CS molecule. Due to the presence of d-glucosamine residues, CS behaves as a polyelectrolyte with a strong positive surface charge in acidic conditions and interacts strongly with polyanionic molecules such as TPP. In neutral and alkaline pH, CS is insoluble unless chemically modified. The first production of CS microparticle via ionotropic gelation was reported by Calvo et al. in 1997 [158], whose approach provided microparticles in the 200–1000 nm range. By the time the study was published, CS beads were already studied for their ability to respond to pH changes and their positive charge, but due to their large size (1–2 mm), they were not suitable for deposition on nasal and mucosal membranes. Also, Calvo’s contribution provided an alternative to the utilisation of glutaraldehyde, a covalent crosslinker that allowed the production of microparticles with a good degree of monodispersity but with possible antigenic effects [173,174]. The procedure offered, for the first time, the possibility of the incorporation of proteins or peptides that would suffer from covalent crosslinking. The study demonstrated that ionotropic gelation prepared CS microparticles allow to encapsulate proteins with high efficiency and laid the foundations for future studies on gene and antigens therapies [158]. Ionotropic gelation has also been extensively studied for the delivery of both small drugs and macromolecules [157]. Despite the apparent simplicity of the process, the interaction of the CS polycation with a polyanion cannot be completely explained by the electro-neutrality principle. The process of gelation involves the formation of a three-dimensional network that occurs due to both the inter and intra-molecular cross linking of positively charged CS chains [157,175]. The TPP anion can possess up to five negative charges depending on the pH of the solution. The crosslink can occur either between two protonated amine groups belonging to the same polymer chains (intra-molecular) or between groups belonging to different polymer chains (inter-molecular) [176]. By changing the preparation parameters, the interaction between CS and TPP can be influenced to produce micro- and nanoparticles and to reduce their dispersity. The fabrication of nanoparticles requires dilute polymer concentrations for local gelation process to take place within small polymer coils. In a semi-dilute regime, the probability of polymer chain overlaps increases, and the formation of much larger particles is most likely to occur. One of the first correlations between CS concentration and the properties of the nanoparticles was reported by Pan et al., although it was based on a limited number of attempts and the use of a single type of CS with high molecular weight (HMW) and an 89% DD [177]. CS particles are generally produced in a concentration range of 0.5 to 2 mg/mL, although some examples of higher concentrations have also been reported [175]. Nevertheless, a direct correlation between the properties of nanoparticles and the properties of CS should be established for each individual batch of CS under consideration. The lack of batch-to-batch uniformity associated with the polymer often results in poor control over size distribution, high dispersity and inconsistent results [178]. In some cases, the associated variables are such that the correlation should be done on a narrow interval of polymer molecular weight. Therefore, it is difficult to guarantee total reproducibility of the experiments. Despite the abundance of empirical data about the preparation of micro- and nanogels through ionotropic gelation, their preparation mainly relies on trial and error and the correlations between their formation process, interaction and structure are not totally understood [157,178,179]. CS chains can originate very different particles based on their structural properties, including molecular weight (MW), index of polydispersity, deacetylation degree (DDA) and d-glucosamine distribution along the polymer backbone. These parameters can affect both the polymer stiffness and flexibility and the viscosity of the solutions used for particle preparation [180]. The most crucial parameter for the formation of particles with low dispersity is the CS/TPP ratio, which defines microgel average diameter and drug-release properties. Lower CS/TPP ratios generally result in smaller particles with lower zeta potential, while higher CS/TPP ratios have been employed to form bigger particles and sometimes to induce the precipitation of micrometric particles. Particles are prepared using a ratio between 1.75:1 and 6:1, although each individual sample needs to be evaluated separately [157,158,175,179,181]. For chemically modified CS, such as N-grafted polymers, these ratios may vary, as ionotropic gelation requires free amine sites to take place [20]. The utilisation of different pH during particle preparation also has a moderate effect on the size of the particles, as pH influences the number of charges on TPP molecules [176]. By decreasing pH, the interaction between CS and TPP is strengthened, and smaller particles are produced. CS nano- and microparticles are prepared in a range of pH between 4.5 and 5.5. According to some studies, the increase in molecular weight has a similar effect as the increase in concentration [178,180,182]. This consideration agrees with the fact that higher molecular weights result in an increase in solution viscosity. Based on this consideration, Zhang et al. prepared particles between 90 and 200 nm by fractionating CS to reduce its molecular-mass distribution. The study showed that particles had low polydispersity (<0.1) compared to other formulations (0.3–0.4), although they were unsuitable for the encapsulation of macromolecules due to reduced molecular weight [182]. Some studies argue that lower molecular weights result in bigger particles due to the reduced flexibility of shorter polymer chains [180]. Other efforts have been made in order to tune the characteristics of CS–TPP nano- and microparticles, including adjusting the salinity of the solvent [157,178], changing temperature [183] and increasing mechanical energy during their preparation [184]. Fan et al. reported a preparation in which the polydispersity (PDI) of microparticles was narrowed to 0.026 by diluting the quantity of acetic acid and reducing the ambient temperature during cross-linking. The microgels had a mean D_h_ of 138 nm and a positive zeta potential. The lower thermal agitation favours the formation of particles in a more orderly way and reduces the overlapping of polymer chains, thus reducing the polydispersity of CS particles [183]. Other studies point out that the size and polydispersity of CS particles can be reduced using monovalent salt solutions [157,178]. Huang et al. demonstrated that the strength of CS/TPP interactions could be strengthened adding a monovalent salt, such as NaCl [178]. Small amounts of salt (150 mM) enhance the colloidal stability of microgels during their formation, while binding is weakened at high ionic strength (500 mM). This suggested the hypothesis of an optimal concentration for the preparation of CS/TPP microgels with narrow size distribution. The hypothesis of Huang et al. was that the presence of NaCl inhibits the bridging of the microgels and prevents their aggregation, demonstrating that CS microgels behave differently than other colloidal systems [178]. Colloidal stability is determined by the potential energy sum of the attractive van der Waals forces and the repulsive electrostatic interactions. This description is the basis of the DLVO theory and is considered valid for the description of the behaviour of inorganic particles in solution [185]. However, when the particles in consideration are fully or partially covered with polymers, their behaviour is more complex. If the polymer layer on the colloid exceeds a minimum thickness, dispersion forces are unimportant. At moderate surface coverage, a critical point exists and depends on the polymer theta point. In the case of CS nano- and microgels, it can be roughly assumed that the microgel behaviour is similar to that of a particle entirely covered with a polymer. Otherwise, it could be imagined that the behaviour of a microgel resembles the one of a “hollow” polymer particle exclusively formed by the polymeric coverage. Therefore, microgels should not be considered “nanoparticles” in the same way as we consider inorganic nanoparticles. Inside the microgel, the polymer chains can move within a series of constraints imposed by the three-dimensional structure of the gel, unlike inorganic particles wherein all the atoms are condensed on the surface of a nanoparticle. Given the size of the only CS macromolecules in solution, which can reach up to 20 nm for high molecular weights, we can assume that small CS aggregates (<100 nm) are made of few macromolecules and their behaviour can be close to that of a single macromolecule in solution. The aqueous behaviour of CS microgels has a direct impact on the release profile. Drug release occurs mainly through three mechanisms: diffusion, swelling and erosion (Figure 3). The type of mechanism is strictly dependent on type (ionic, covalent) and degree of crosslinking. Covalent cross-linked microgels, such as CS-glutaraldehyde [186], usually provide diffusion-controlled drug release, with the overall release profile depending on the cross-linking degree. Similarly, swelling capacity is influenced by cross-linking density and environmental pH [187]. The utilisation of ionic cross-linkers, such as TPP, may result in erosion release profiles. In the initial part of the release profile, the kinetics of swelling and erosion determines a characteristic lag phase. Model equations for the interpretations of several different studies have been proposed by a huge number of studies [188,189,190]. Diffusion-controlled behaviour can be interpreted with exponential functions, and erosion-controlled release curves have a characteristic sigmoidal shape.

## 5. Chitosan-Poly(*N*-vinylcaprolactam) (CP) Microgels for Thermoresponsive Drug Delivery Systems

The poor biodegradability of thermoresponsive polymers, such as PNIPAM and PNVCL represents the main limit for their biological application [24,168,191]. The solution of this problem was provided by the development of hybrid materials that were prepared by combining synthetic thermoresponsive polymers with biopolymers of natural origins [16,192,193,194], such as CS. The synthesis of these hybrid materials has led to the development of thermoresponsive and biodegradable devices for drug delivery, such as gels and particles in the nano-, micro- and macroscale. Among various natural biopolymers that can be combined with a synthetic thermoresponsive polymer, CS has proven to be useful and versatile for its high compatibility and pH-responsiveness.

The preparation of the first hydrogel that combined PNIPAM and CS was reported by Wang et al. in 2000 [186]. The gel exhibited thermoresponsivity, and the LCST was determined to be 32 °C, close to the LCST of the starting PNIPAM (31 °C). For the preparation of CS-based thermoresponsive gel, PNIPAM was modified with carboxyl termination groups prior to the preparation of the gel, and gelation was achieved using glutaraldehyde [186]. Thermoresponsive polymers with carboxyl group terminations represent the most common way to prepare CS thermoresponsive copolymers. These pre-modified polymers are generally referred to as -COOH polymers (e.g., PEG-COOH, PNVCL-COOH, PNIPAM-COOH). The carboxyl end groups allow the creation of an amide bond with the amine sites present on d-glucosamines distributed along the skeleton of CS. The coupling between the two functional groups results in a graft polymer formation. The reaction is commonly carried out through the activation of carboxyl end group using NHS and EDC. The initial procedure reported by Wang et al. did not require the synthesis of a copolymer prior to gelification.

**Figure 4:**
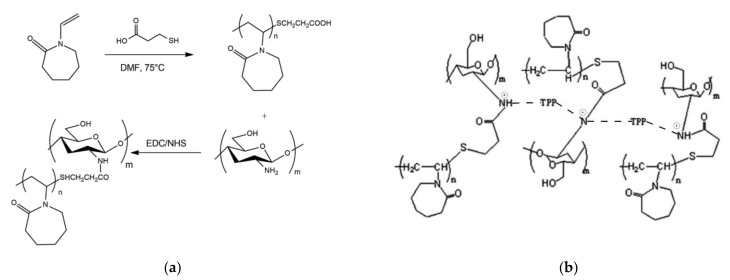
(**a**) Reaction scheme for the synthesis of chitosan-*graft*-poly(*N*-vinylcaprolactam). (**b**) Possible mechanisms for the ionic crosslinking of chitosan-*graft*-poly(*N*-vinylcaprolactam) microgels, as described by Rejinold. Adapted with permission from Rejinold [16], copyright by Elsevier, 2011.

The synthesis of a thermoresponsive CS-g-PNIPAM copolymer was later described in 2006 by Chen et al. [195], while the first CP copolymer (CP) was prepared by Prabaharan et al. in 2008 [14] (Figure 4a). Numerous types of graft-copolymers have been prepared with CS using the coupling reaction between amino and carboxyl groups. This type of reaction falls within the field of click chemistry, as it is relatively fast, requires mild conditions and produce inoffensive byproducts. Conversely, the preparation of CS copolymers with different architectures (e.g., star, comb, brush, ring block) requires complex and expensive synthetic procedures [196]. Also, the utilisation of CS as a reagent entails some limitations for multi-step reactions due to its extremely low solubility in organic solvents. Due to the huge number of functional groups, CS chemical modification is usually time-consuming. Depending on the mass of the polymer, the initial protection reaction of the functional groups not involved in the reaction can take up to several days, depending on the molecular mass of CS [194].

Similarly, chemical structure determination through simple spectroscopic techniques, such as ^1^H-NMR, can become very complex due to long-lasting spectral acquisition. Although a single polymer ^1^H-NMR spectrum with sufficient resolution can be acquired within a single day, the acquisition of a ^13^C-NMR spectrum or a two-dimensional spectrum may take up to one week due to the high number of functional groups and the viscosity of the solutions [197]. Therefore, these synthetic procedures are rarely accomplished inside university facilities, and they are carried out on a larger scale by research institutes specialised in the study and the development of few specific polymers. For simplicity, scientists have turned their attention to the utilisation of LMW CS with low dispersity for synthetic purposes [157,180]. Also, the utilisation of LMW CS for drug delivery systems has been promoted for its better solubility, biocompatibility, bioactivity, biodegradability and less toxicity in relation to HMW CS [183]. Nevertheless, the mild conditions required for the formation of graft copolymers allow the utilisation of both LMW and HMW CS for the preparation of thermoresponsive copolymers. This allows the maintenance of some interesting biological properties connected to polysaccharide’s high molecular weight. Both LMW and HMW CS are highly regarded for drug delivery, but only HMW have shown high encapsulation efficacy towards proteins [182,198]. Furthermore, HMW CS requires less chemical processing. In relation to LMW CS, HMW CS is cheaper and regarded as a “greener” reagent. To date, CP copolymers have been prepared with both HMW and LMW CS for the release of both hydrophilic and hydrophobic drugs.

The first CP copolymer was prepared using LMW CS (~30 kDa). Prabaharan et al. described CP as promising for possible applications in dual-responsive therapies. Their study reported a protocol for the encapsulation of a hydrophobic drug, ketoprofen, during the preparation of CP beads via ionotropic gelation. The size of the beads was not determined, suggesting that particles were at least micrometric in size. In fact, the term beads is commonly used as an abbreviation for the term microbeads, which are defined as uniform polymeric particles with a size between 0.5 and 500 μm in diameter [199]. The ability of the copolymer beads to respond to both pH and temperature stimuli was demonstrated by swelling studies and release tests [14]. The cytotoxicity of CP beads was assessed using an MTT assay against EA.hy 926 endothelial cell lines and researchers demonstrated that CP would have provided a safe and effective drug-delivery carrier within the living body. Despite not being nanotechnology-related research, the work of Prabaharan et al. paved the way for the development of CP-based nanoformulations [12,16,18,20,192]. However, the discussion on the behaviour of PNVCL-COOH by Prabaharan et al. remains controversial. In its introduction, Prabaharan described PNVCL as a “*well-studied polymer with a lower critical solution temperature (LCST) at about 32 °C, which shows a well-defined response towards temperature*” [85] while not considering that PNVCL is a type I thermoresponsive polymer. In fact, PNVCL can exhibit a LCST between 25 and 50 °C, as it depends on PNVCL molecular weight, concentration and the presence of salts. Moreover, the characterisation of PNVCL-COOH in GPC-SEC was carried out in THF [14], a solvent later described as unsuitable for the molecular determination of PNVCL [24,200]. Furthermore, few technical details on the analysis were reported. The molecular mass, established to be around 1 kDa [14], appears to be too low in relation to the LCST value. The data disagreed with the LCST-molecular-mass dependence previously reported by Meeussen [144,145], Kirsh [201] and, later, by Cortez-Lemus [24]. According to Meeuseen’s predictions, PNVCL must be at least 40 KDa to exhibit LCST lower than 35 °C [145,202], even though the Meeusen study did not account for PNVCL with carboxyl group terminations. Although it is known that the presence of terminations of compounds that enhance the hydrophilicity of PNVCL are known to increase the LCST of the polymer [203,204], the article did not provide an explanation for such a huge difference in the molecular mass values related to the LCST. The molecular-weight value reported by Prabaharan corresponds to a small oligomer made of 8 repetitive units of NVCL. This seems apparently unreasonable considering the high molar ratio (122:1) used for the synthesis of PNVCL-COOH [14]. Despite the lack of insight about the miscibility behaviour of PNVCL-COOH, the following works on the development of CP-based delivery systems seem to neglect the problematic aspects of Prabaharan’s work [12,16,18,20,192,202].

The work of Prabaharan on CP-related systems was continued by the research group of Indulekha et al. Instead of reporting the procedure for the synthesis of PNVCL-COOH, Indulekha referred directly to the preparation previously reported by Prabaharan [12,18,20]. Other authors reported a very similar procedure for the synthesis of a PNVCL-COOH polymer with a LCST of 32 °C [16,192]. None of these studies mentioned the different chemical–physical properties that exist between PNVCL polymers of different lengths, nor did they provide detailed guidance for the characterisation of their molecular mass. These works have contributed to passing down the misconception that PNVCL possesses a well-defined LCST at 32 °C. Due to the difficulty of replicating the results of the published works, the research on CP-related nanodevices remained confined to a few research groups (Table 1).

The first CP-related study within the nanotechnology field was published by Rejinold in 2011 [192]. It described a protocol for the preparation of microparticles for the release of 5-fluoruracil. Since 5-fluoruracil is a chemotherapeutic, the study opened up the possibility of using CP of materials for oncological applications. Rejinold reported that CP polymer was prepared with different ratios between CS and PNVCL-COOH in order to achieve a desired transition temperature of 38 °C for the drug release. The LCST of the copolymer increased as the percentage of thermoresponsive polymer decreases. Drug encapsulation was achieved by synthesising nanoparticles by dissolving the drug into a concentrated CP solution (5 mg/mL). The synthesis of microparticles was achieved via ionotropic gelation (Figure 4b). Acetic acid concentration was rather high (1%) compared to what has been reported for the preparation of monodisperse CS nanoparticles [183]. The average D_h_ of the particles was reported to be between 180 and 220 nm, and the sample polydispersity was not reported. The complete distribution curve between 1 and 4000 nm is not available in the publication. Instead, the histogram related to the distribution focused only on the interval 100 and 300 nm. The main peak of the histogram is at 1% intensity, suggesting that the sample could have been highly polydisperse. We calculated that the integration of the size range provided by Rejinold provides a total percentage inferior to 5% of the reported intensity. It may be thought that the main peak of the distribution was located at higher values. Also, the number of measurements reported (3) is too low compared to the average standard of DLS analyses. A DLS analysis using a Malvern Z-Sizer, one of the most common instruments for the determination of the D_h_, requires three cycles of analysis, each of which includes between 10 and 17 measurements. CP particles were purified by centrifugation and lyophilised using sucrose as a cryoprotectant. Release tests were carried out by the lyophilised particles in phosphate buffer (pH 7.4) and show a dramatic change in the behaviour of CP particles in relation to temperature. Despite the promising results of this research group, there is not enough evidence to establish that the elution profile of a CP nanoparticle is similar to that of the lyophilised particles described by Rejinold.

In another work published in the same year, Rejinold proposed a similar preparation for the preparationof curcumin-loaded CP microgels [16]. CP particles undergo thermal-induced aggregation at 38 °C, and their blood compatibility was demonstrated via a haemolysis assay. Curcumin-loaded microgels showed specific toxicity to cancer cells at above their LCST, and the analysis of the JC-1 mitochondrial membrane confirmed that the apoptosis was mitochondrial-mediated [16].

A few years later, the same research group introduced for the first time the synthesis of CP hybrid particles consisting of CP microgels loaded with both Au NPs and curcumin [142]. Microgels were prepared using ionotropic gelation, by mixing Au NPs with the copolymer prior to gelation. The study was based on the premise that Au-NPS being heated via the exposure of radiofrequencies (RF). Since Au-NPs are RF-heatable, the release of encapsulated curcumin from the microgels was induced by the presence of Au-NPs at optimum RF conditions. CP microgels had an average D_h_ of 160 ± 20 nm and a positive surface charge and showed excellent and selective efficacy towards breast cancer cells and enhanced circulation and biodistribution in relation to free curcumin. For anti-cancer assessment, MCF-7 and T47D cells were used. Cells were washed with metal-free solutions and maintained under a RF chamber at 40 W for 5 min. The samples exposed to RF showed higher apoptosis in relation to that without exposure. The in vivo test on 5–6 weeks old nude mice demonstrated the CP microgels were retained at the colon tumour for 2 weeks [142]. The preparation of CP microgels loaded with Au NPs and curcumin was firstly proposed in another paper that was later retracted. The process was based on the synthesis of Au NPs of 10, 20 and 50 nm in the presence of starch and D-glucose. However, the details for the preparation of Au NPs of a specific diameter were not provided. Furthermore, there was no information related to the size of Au NPs used for the preparation of CP microgels [142]. In 2016, Rejinold reported the first preparation of a hybrid system consisting of CP particles and Fe_3_O_4_ nanoparticles for the release of curcumin. Magnetic nanoparticles and the drug were added to CP solutions prior to ionotropic gelation. Similarly, indocyanine green and rhodamine-123 were added to the solutions for labelling purposes, as previously reported for the preparation of Au-CP NPs [142]. The hybrid particles were able to respond to the application of an alternating magnetic field and were tested in vitro on 4T1 breast cancer cells and in vivo on normal Swiss albino mice. CP-hybrid microgels possessed an average D_h_ of 180 ± 20 nm and showed cellular internalisation on 4T1 breast cancer cells and radiofrequency (RF)-dependent curcumin release in vitro. The exposure to RF induced local heating within CP microgels, causing the drug release. The in vivo studies demonstrated the feasibility of the system as nanotherapeutics for the treatment of breast tumours, as CP particles prolonged the circulation of curcumin and showed significant tumour localisation. A similar work was published by Indulekha in 2017, which also featured the contribution of Prabaharan. In their study, pH-, temperature- and RF-responsive particles were prepared using microgels of CP loaded with DOX and Fe_3_O_4_ nanoparticles for the treatment of breast cancer cells. CP particles were prepared accordingly, simply mixing the magnetic nanoparticles and DOX with CP polymer before gelation. CP microparticles were purified through dialysis against distilled water. The study showed the different behaviour of microparticles in relation with different pH and temperature conditions and under alternating magnetic field exposure. The application of an alternating magnetic field resulted in a step-like elution profile of the chemotherapy drug. This study represents the first example of the remote control of the elution of a CP-based drug nanoformulation. Compared to the previous works by Rejinold, Indulekha’s research team reported a more complete description of the size distribution of CP microgels and showed the change of the D_h_ of CP microgels as function of temperature. It can be observed that above the critical temperature (reported as LCST), the average D_h_ is shifted to higher values. This may suggest that temperature-induced drug release is provoked by the swelling of CP microgels, as was hypothesised by Rejinold [192], but the drug-release mechanism is not described. Although it is evident that the change of pH or temperature accelerates drug release, the similar profiles of the release curves suggest that the release dynamics do not change during thermo-induced transition. This work, as well as the previous ones, does not distinguish between the thermoresponsive behaviour of the polymer (LCST) and that of microgels (VPTT). According to the results of Indulekha, the magnetic-pH-thermoresponsive particles could encapsulate DOX with very high efficiency (∼57%). This value is in disagreement with what was previously reported [209], since it is well-known that DOX interacts very weakly with CS-based polymers. The release behaviour of Indulekha’s particles resembled the release mechanism reported by Rejinold [30]. The absence of the use of a cryoprotector during freeze-drying suggests the hypothesis that release tests were carried out using aggregates formed as a lyophilisation process result instead of the nanoparticles measured with DLS [150]. Furthermore, the size of freeze-dried nanoparticles was not reported in the study [150]. The reduced in vitro toxicity of the encapsulated DOX has been described as a promising element for the utilisation of CP-based devices for the treatment of breast cancer. Indulekha also developed a thermoresponsive transdermal drug delivery system consisting of CP macrogels for the treatment of local pain with an on-demand localised drug delivery system. The drug was tested at three different temperatures (25, 32 and 39 °C) at two different pHs (5.5 and 7) to demonstrate that drug release was enhanced above the polymer LCST (39 °C) in mild acidic pH (5.5). The system was tested for the release of two hydrophobic drugs, acetamidophenol and etoricoxib. The gel biocompatibility was demonstrated by in vivo studies in rat skin [12]. Again, Indulekha reported a preparation of microgels of CP loaded with Au NPs for the treatment of breast cancer with photothermal therapy. Although the utilisation of Au NPs with CP microgels for breast cancer treatment was already reported by Rejinold, Indulekha’s work had a completely different approach and provided an accurate characterisation of Au-CP hybrid nanodevices. Indulekha used bigger Au NPs prepared in the presence of a CP dispersion. Au NPs were nucleated directly on the surface of CP microgels using ascorbic acid as a reducing agent, as suggested by both UV-VIS and TEM characterisations. Due to their “*ruffled*” morphology, Indulekha improperly used the term “core” and “shell” in reference to the morphology of microgels. Their behaviour was similar to core-shell nanoparticles, and CP microgels exhibited a tuneable absorption in relation to the ratio between the amount of CP and Au NPs. This allowed the tuning of the device to ensure absorption in the NIR (750 nm) frequencies. Cytotoxicity tests on normal mouse fibroblast L929 showed a substantial reduction in the toxicity of Au NPs. CP-Au devices were demonstrated to be biocompatible with both L929 and human breast adenocarcinoma cells MC-F. The exposition to a 750 nm laser reduced the viability of MCF-7 cells from 90% to approximately 5%. Furthermore, the study demonstrated that the nanodevice could be used as a biocompatible X-ray contrast agent. In relation to commercial iodine-based Omnipaque, Au/CP microgels give greater contrast and require less-concentrated samples due to the high electron density of Au. Thus, the study suggested the utilisation of an Au-CP based microgel disintegrable theranostic nanoprobe for image-guided triple therapy consisting of photothermal, chemo and radiotherapy treatment [18].

More recently, other CP-related systems have been developed, and CP polymers have started to show some promising properties for environmental applications. In 2019, Bahmani developed CP/ZIF-8 (zeolitic imidazolate framework) nanofibers to remove As (V) and Cr(VI) from aqueous solutions [210]. In 2021, a tri-block polymer PAA, PNVCL and CS was developed and used as a biocompatible flocculant for water remediation [211]. The polymer provided an excellent device for the removal of turbidity, ciprofloxacin and Cd(II) from aqueous solutions, and its ability to bind pollutants increased above the LCST. The tri-block polymer was prepared by polymerising acrylic acid in the presence of CP [211]. The synthesis of another tri-block polymer is reported by Durkut, who prepared a biocompatible polymer of CS-g-galactosilate-g-PNVCL that shows pH- and temperature-dependent responsivity [4]. The polymer was tested for bovine serum albumin (BSA). In the field of nanotechnology, some authors have reported the synthesis of other hybrid nanodevices based on polymeric and inorganic nanoparticles. Niu and his coworkers reported a brilliant strategy for the preparation of a CP-peptide self-assembled nanoformulation for DOX release for breast cancer treatment [205]. The study reported a multi-step synthesis of the CP-peptide involving the protection of amine residues through phtaloylation [196]. The synthesis was carried out with an LMW CS (10 kDa). NVCL was conjugated to CS by reversible addition fragmentation, chain transfer was then conjugated (RAFT) polymerisation using S-1-dodecyl-S′-(α,α′-dimethyl-α″-acetic acid) trithiocarbonate (DDACT). Thus, after the removal of phthaloyl residues, amide residues were used to conjugate a peptide that allowed the device to selectively recognise MCF-7 breast tumour cells. Unlike the previous work, the particles were not formed through ionotropic gelation. Nia reported that the polymer self-assembled in aqueous solutions in ~200 nm microparticles. The results in vivo and in vitro on MCF-7 cells and xenografted mice demonstrated that the microparticles were taken up by cancerous cells with a substantial reduction of DOX toxicity and a significant reduction in tumour volume that resulted in a prolongation of lifespan [205]. In 2020, Banihashem published a work similar to those of Indulekha [18] and Rejinold [142], that reported the preparation of another Au/CP nanocarrier for the treatment of MCF cells with cisplatin [208]. This work introduced the hypothesis that the utilisation of thioglycolic acid as a ligand for Au NPs stabilisation may strengthen the interaction between CP and Au NPs during ionotropic gelation. Baninashem also reported a study for the development of CS nanofibers coated with Au-Au sulphide nanoparticles [207]. A new method for the preparation of CP-Fe_3_O_4_ was reported by Sahebi [206]. In his work, a polymer is prepared through a step synthesis involving the polymerisation of NVCL in the presence of Fe_3_O_4_ nanoparticles. The PNVCL-capped NPs were subsequently conjugated to CS with EDC/ NHS. The hybrid magnetic particles were used for the selective recognition of imatinib mesylate from biological samples.

## 6. The Role of Polymer–Drug Interaction: The Example of Doxorubicin and Chitosan

To ensure the proper functioning of a controlled-release drug delivery system, it is necessary to know the interaction between drug and particle. One of the main problems related to the use of particles prepared by ionotropic gelation is the absence of covalent bonds between the drug and the CS particle. On the contrary, the presence of weak interactions ensures the greater flexibility of microgels but, at the same time, does not guarantee the retention of the drug in physiological conditions. In this section, we will focus on the interaction between CS and doxorubicin (DOX), a hydrophilic drug that is commonly used in cancer treatments.

**Figure 5:**
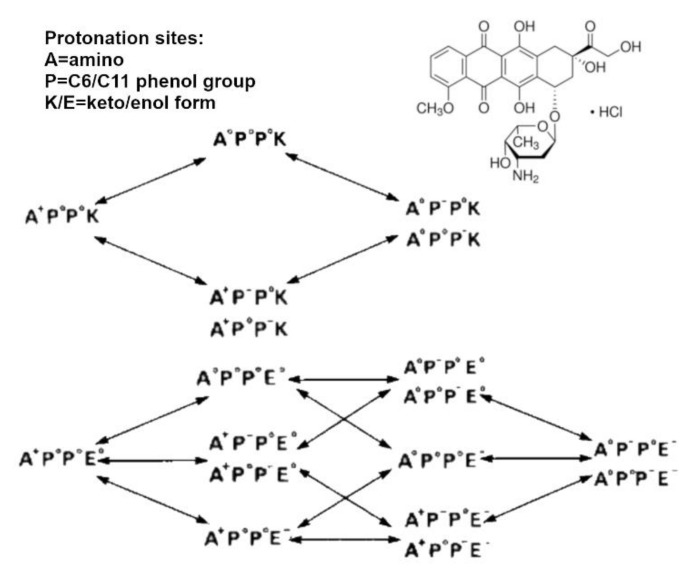
Protolytic equilibria of doxorubicin between pH 1 and 12. Adapted from Beijnen [218], copyright by Elsevier, 1986.

Doxorubicin (DOX), also known as Adriamycin, is an anthracycline that belongs to the family of anticancer antibiotics. The molecule can be described as a tetracenequinone with a sugar attached by a glycosidic bond. Due to the tetracyclic structure of anthraquinone, the molecule can intercalate between two DNA bases, while the sugar enters the minor groove and interacts with the adjacent base pair. Within its structure, DOX has five hydroxyl groups, two phenolic and three alcoholic, and one amino group. Consequently, DOX is slightly soluble in water and is a weakly acidic compound. The estimated values for pKas are 7.34 (phenolic group), 8.46 (amino group) and 9.46 (source: Sparc), respectively. These values indicate that DOX exists in cationic form in a pH range between 5 and 9. In aqueous solution, DOX molecules tend to self-associate via π–π interactions [212] and form gel-type structures. In addition, DOX chelates strongly di- and trivalent ions [213] and is subject to photolytic decomposition [214,215,216] and oxidation [217]. The use of DOX in chemotherapy has therefore placed the need to develop pharmaceutical formulations that increase both drug solubility and effectiveness. In particular, DOX in aqueous solution undergoes conversion to doxorubicinone and daunosamine [218]. This process is initiated by a tautomeric equilibrium and is catalysed by the presence of buffers and the increase of temperature. According to a study by Beijnen et al., the best condition to stabilise a DOX solution is by using a diluted acetate buffer at pH 4.00, while conversion constants increase dramatically for pH above 6 or below 3 [218,219]. CS solutions are also generally prepared in low-concentration acetate buffers (1% *w*/*v*) in a pH range between 4 and 5.5. Since CS and DOX are prepared in similar conditions, many studies focused on the development of DOX-delivery systems based on CS-based polymers [20,27,205,209,220,221]. Nonetheless, the interaction between the two compounds is difficult to explain due to the complexity of the equilibria that arise in solution. In a pH range between 4 and 6, it is necessary to consider that the DOX molecule can exist in neutral or zwitterionic form and that each type can undergo a degradation process catalysed by the solvent or the presence of protons in solution [218,219] (Figure 5).

At pH 4.00, since the pH is well below the pKa of the first phenolic dissociation (7.34), it can be assumed that both DOX and CS behave as cations. The overall interaction between the two species is influenced by the DDA of CS, as the presence of deacetylated residues lowers the positive surface charge of the polymer, thereby decreasing the ionic repulsion. Accordingly, the encapsulation of DOX in a CS nanoparticle would require the chemical modification of the structure of the drug or polymer [164]. Alternatively, non-modified DOX can be trapped by dissolving DOX in a different phase, and the encapsulation is achieved via the formation of emulsions or microemulsions [222]. During ionotropic gelation, DOX can be entrapped by the formation of the microgel cage around them. Eventually, DOX can elute outside the three-dimensional network of the CS gel into the external solution. In this condition, the drug requires a strong driving force in order to remain anchored inside the cavity of the CS microgel. In 2001, only four years after the development of ionotropic-gelated CS NPs by Calvo et al. [181], a study by Janes et al. already pointed out that CS-based particles were a poor substrate for DOX encapsulation [209]. The study reported that the DOX loading efficiency of non-modified CS NPs could not exceed 2%. Nevertheless, CS NPs has been widely used for the encapsulation of DOX, and some studies reports loading efficacy higher than 50% [20,205]. However, these promising results are rarely accompanied by an accurate description of the encapsulation phenomenon [12,18,20,27]. In a few cases, some of these studies were retracted from publication journals. In fact, one of the possible mistakes of the authors may be related to the utilisation of a pharmaceutical formulation of DOX, such as Adriamycin. DOX standards are generally expensive and pharmaceutical formulations are often used, even if they may contain excipients and other components, such as methyl 4-hydroxybenzoate (methylparaben), which prevents self-association between DOX molecules. For instance, lyophilised Adriamycin powder contains methylparaben and lactose, and the mass percentage of DOX corresponds to about 10% of the formulation. In this case, if the encapsulation efficiency is calculated via UV-VIS, the calibration curve must be based on the extinction coefficient of the pharmaceutical formulation of the drug. In general, DOX content in water is estimated by using an extinction coefficient between 10,410 [20] and 125,000 L mol^−1^ cm^−1^ [223]. There is a possibility that some studies did not consider that the extinction coefficient value is affected by the solvent. In a study by Etrich et al. [224], the aqueous content of DOX was determined by using the value of the extinction coefficient of DOX calculated in ethyl acetate by Subr et al. [225]. Although inaccurate, such values may be close to those that are expected. However, the utilisation of drug formulation can lead to the strong overestimation of DOX encapsulation efficacy (EE). EE is calculated from the absorbance value of the solution, so that lower values of absorbance correspond to higher encapsulation values. If not properly calibrated, the value can be overestimated. According to the previous example of lyophilised Adriamycin, since the content of DOX corresponds to only 10% of the product, a non-calibrated estimation would lead to the conclusion that the encapsulation efficacy is ten times higher than its real value. If not correctly calibrated, the value can be strongly overestimated. A brief explanation of the possible interaction between DOX and CS was provided by Sadighian et al. [27]. DOX can interact with CS only via weak forces, such as hydrogen bonds and electrostatic interactions. DOX and CS interact by a polar “imine bond” that is sensitive to pH, which is developed between the carbonyl group of DOX and the amino group of CS. According to this hypothesis, CS NPs can retain DOX for few days [27]. In another study by Sanyakamdhorn et al., the overall (non-covalent) interaction between CS and DOX was studied using molecular modelling [220]. According to reported results, the overall interaction was −3.89 kJ/mol for a CS made of 19 repeating units (about 3.4 KDa). Consequently, the overall interaction between an oligomer of CS and DOX is weaker than a single hydrogen bond. The study also suggested that CS with higher molecular masses are better candidates for DOX encapsulation. Finally, another possibility is that the utilisation of aggregated CS NPs increases the EE of DOX. Many studies do not mention the procedural difficulties associated with the lyophilisation of CS particles [16,18,20,27,30]. Since the characterisation of CS NPs is reported prior to freeze-drying process, they provide no evidence about the structure or size of the freeze-dried particles. However, as reported by Rampino et al. [150], the lyophilisation of CS NPS require treatment with cryoprotectors, and freeze-dried particles usually exhibit different size, surface charge and morphology. The differences between the chemico-physical properties of CS NPs and lyophilised CS NPs can have a strong influence on the release mechanism of the drug.

## 7. Conclusions and Future Perspectives

To date, thanks to their multi-responsivity, CP-based polymers and their hybrid derivatives represent a novelty for the development of cascade-responsive delivery systems for oncological applications. Despite the promising results obtained during in vitro and in vivo experimentations on xenografted mice, there are many aspects that still need to be clarified in order to expect a clinical translation of these studies. The reproducibility of the fabrication of such delivery devices can be improved by providing a detailed analysis of how the structural properties of PNVCL and CP affect the thermoresponsivity of polymers and particles. Furthermore, further studies are needed to confirm the swelling-mediated drug release of CP microgels and there is only little information about drug uptake mechanism during their preparation. The preparation of CP-delivery systems requires a better understanding of the lyophilisation protocols of microgels and of the mechanism of interaction between the drugs and the CS particles. For clinical applications, it is fundamental to investigate how mechanical and chemical-physical properties of microgels are modified by lyophilisation, as well as the ability of freeze-dried particles to maintain properties over time. In the future, we may expect that CP-based systems will also find their application in the same fields in which CS-based systems have already been used, such as ocular delivery systems and the treatment of prostate and liver cancer (HCC).

## Figures and Tables

**Table 1:** List of publications involving chitosan-*graft*-poly(*N*-vinylcaprolactam) polymeric systems for drug delivery applications.

Target	Drug	Delivery System	Trigger	Year	Ref.
human endothelial cell line	ketoprofen	Micrometric beads	pH	2008	[14]
			Temperature		
PC3 and L929 cells	curcumin	Microgels	pH	2011	[16]
			Temperature		
MCF 7 and L929 cells	5-fluorouracil	Microgels	pH	2011	[192]
			Temperature		
L929, MCF 7 and T47D cells	curcumin	Microgels + Au NPs	pH	2015	[142]
			Temperature		
			RF		
abdominal skin (SD rats)	acetamidophenol	Hydrogels	pH	2016	[12]
	etoricoxib		Temperature		
4T1 cells and Balb/c mice	curcumin	Microgels + magnetic NPs	pH	2016	[30]
			Temperature		
			RF		
MCF 7 and MDAMB 231 cells	doxorubicin	Microgels + magnetic NPs	pH	2017	[20]
			Temperature		
			RF		
MCF 7 cells	-	Microgels + Au NPs	pH	2017	[18]
			Temperature		
			NIR		
HDF cells	BSA	Microgels	pH	2018	[4]
			Temperature		
TNB xenograft mouse	doxorubicin	Nanocomposite	pH	2019	[205]
			Temperature		
tyrosine kinase inhibitor treatments	imatinib	CP-coated magnetic NPs	pH	2019	[206]
			Temperature		
			RF		
MCF 7 cell lines	Cisplatin	Nanofibers + Au NPs	pH	2020	[207]
			Temperature		
MCF 7 cell lines	cisplatin	CP-coated Au NPs	pH	2020	[208]
			Temperature		

## Data Availability

The data presented in this study are available on request from the corresponding author.

## References

[1] Kawaguchi H. (2014). Thermoresponsive microhydrogels: Preparation, properties and applications. Polym. Int..

[2] Oh J.K., Drumright R., Siegwart D.J., Matyjaszewski K. (2008). The development of microgels/nanogels for drug delivery applications. Prog. Polym. Sci..

[3] McNaught A.D., Wilkinson A. (2007). Definitions of terms relating to the structure and processing of sols, gels, networks, and inorganic-organic hybrid materials. IUPAC. Compendium of Chemical Terminology.

[4] Durkut S. (2019). Thermoresponsive poly (*N*-vinylcaprolactam)-g-galactosylated chitosan hydrogel: Synthesis, characterization, and controlled release properties. Int. J. Polym. Mater. Polym. Biomater..

[5] Kean T., Thanou M. (2010). Biodegradation, biodistribution and toxicity of chitosan. Adv. Drug Deliv. Rev..

[6] Duong H.T.T., Hughes F., Sagnella S., Kavallaris M., MacMillan A., Whan R., Hook J., Davis T.P., Boyer C. (2012). Functionalizing biodegradable dextran scaffolds using living radical polymerization: New versatile nanoparticles for the delivery of therapeutic molecules. Mol. Pharm..

[7] Yin Y., Hu B., Yuan X., Cai L., Gao H., Yang Q. (2020). Nanogel: A Versatile Nano-Delivery System for Biomedical Applications. Pharmaceutics.

[8] Fernández-Quiroz D., González-Gómez Á., Lizardi-Mendoza J., Vázquez-Lasa B., Goycoolea F.M., San Román J., Argüelles-Monal W.M. (2015). Effect of the molecular architecture on the thermosensitive properties of chitosan-g-poly(*N*-vinylcaprolactam). Carbohydr. Polym..

[9] Navya P.N., Kaphle A., Srinivas S.P., Bhargava S.K., Rotello V.M., Daima H.K. (2019). Current trends and challenges in cancer management and therapy using designer nanomaterials. Nano Converg..

[10] Gandhi A., Paul A., Sen S.O., Sen K.K. (2015). Studies on thermoresponsive polymers: Phase behaviour, drug delivery and biomedical applications. Asian J. Pharm. Sci..

[11] Schmaljohann D. (2006). Thermo- and pH-responsive polymers in drug delivery. Adv. Drug Deliv. Rev..

[12] Indulekha S., Arunkumar P., Bahadur D., Srivastava R. (2016). Thermoresponsive polymeric gel as an on-demand transdermal drug delivery system for pain management. Mater. Sci. Eng. C.

[13] Tian Y., Bromberg L., Lin S.N., Alan Hatton T., Tam K.C. (2007). Complexation and release of doxorubicin from its complexes with pluronic P85-b-poly(acrylic acid) block copolymers. J. Control. Release.

[14] Prabaharan M., Grailer J.J., Steeber D.A., Gong S. (2008). Stimuli-responsive chitosan-graft-Poly(*N*-vinylcaprolactam) as a promising material for controlled hydrophobic drug delivery. Macromol. Biosci..

[15] Park W., Chen J., Cho S., Park S.J., Larson A.C., Na K., Kim D.H. (2016). Acidic pH-Triggered Drug-Eluting Nanocomposites for Magnetic Resonance Imaging-Monitored Intra-Arterial Drug Delivery to Hepatocellular Carcinoma. ACS Appl. Mater. Interfaces.

[16] Sanoj Rejinold N., Muthunarayanan M., Divyarani V.V., Sreerekha P.R., Chennazhi K.P., Nair S.V., Tamura H., Jayakumar R. (2011). Curcumin-loaded biocompatible thermoresponsive polymeric nanoparticles for cancer drug delivery. J. Colloid Interface Sci..

[17] Swamy B.Y., Chang J.H., Ahn H., Lee W.K., Chung I. (2013). Thermoresponsive N-vinyl caprolactam grafted sodium alginate hydrogel beads for the controlled release of an anticancer drug. Cellulose.

[18] Chauhan D.S., Indulekha S., Gottipalli R., Reddy B.P.K., Chikate T.R., Gupta R., Jahagirdar D.N., Prasad R., De A., Srivastava R. (2017). NIR light-triggered shrinkable thermoresponsive PNVCL nanoshells for cancer theranostics. RSC Adv..

[19] Wust P., Hildebrandt B., Sreenivasa G., Rau B., Gellermann J., Riess H., Felix R., Schlag P. (2002). Hyperthermia in combined treatment of cancer. Lancet Oncol..

[20] Indulekha S., Arunkumar P., Bahadur D., Srivastava R. (2017). Dual responsive magnetic composite nanogels for thermo-chemotherapy. Colloids Surf. B Biointerfaces.

[21] Luckanagul J.A., Pitakchatwong C., Ratnatilaka Na Bhuket P., Muangnoi C., Rojsitthisak P., Chirachanchai S., Wang Q., Rojsitthisak P. (2018). Chitosan-based polymer hybrids for thermo-responsive nanogel delivery of curcumin. Carbohydr. Polym..

[22] Wedel B., Zeiser M., Hellweg T. (2012). Non NIPAM based smart microgels: Systematic variation of the volume phase transition temperature by copolymerization. Z. Phys. Chem..

[23] Cazares-Cortes E., Espinosa A., Guigner J.M., Michel A., Griffete N., Wilhelm C., Ménager C. (2017). Doxorubicin Intracellular Remote Release from Biocompatible Oligo(ethylene glycol) Methyl Ether Methacrylate-Based Magnetic Nanogels Triggered by Magnetic Hyperthermia. ACS Appl. Mater. Interfaces.

[24] Cortez-Lemus N.A., Licea-Claverie A. (2016). Poly(*N*-vinylcaprolactam), a comprehensive review on a thermoresponsive polymer becoming popular. Prog. Polym. Sci..

[25] Nayak S., Gan D., Serpe M.J., Lyon L.A. (2005). Hollow thermoresponsive microgels. Small.

[26] Pitakchatwong C., Chirachanchai S. (2017). Thermo-Magnetoresponsive Dual Function Nanoparticles: An Approach for Magnetic Entrapable-Releasable Chitosan. ACS Appl. Mater. Interfaces.

[27] Sadighian S., Rostamizadeh K., Hosseini M.J., Hamidi M., Hosseini-Monfared H. (2017). Magnetic nanogels as dual triggered anticancer drug delivery: Toxicity evaluation on isolated rat liver mitochondria. Toxicol. Lett..

[28] May J.P., Li S.D. (2013). Hyperthermia-induced drug targeting. Expert Opin. Drug Deliv..

[29] Hervault A., Thanh N.T.K. (2014). Magnetic nanoparticle-based therapeutic agents for thermo-chemotherapy treatment of cancer. Nanoscale.

[30] Rejinold N.S., Thomas R.G., Muthiah M., Lee H.J., Jeong Y.Y., Park I.K., Jayakumar R. (2016). Breast tumor targetable Fe_3_O_4_ embedded thermo-responsive nanoparticles for radiofrequency assisted drug delivery. J. Biomed. Nanotechnol..

[31] Jaiswal M.K., Banerjee R., Pradhan P., Bahadur D. (2010). Thermal behavior of magnetically modalized poly(*N*-isopropylacrylamide)-chitosan based nanohydrogel. Colloids Surf. B Biointerfaces.

[32] Kumar A., Jena P.K., Behera S., Lockey R.F., Mohapatra S., Mohapatra S. (2010). Multifunctional magnetic nanoparticles for targeted delivery. Nanomed. Nanotechnol. Biol. Med..

[33] Patra J.K., Das G., Fraceto L.F., Campos E.V.R., del Pilar Rodriguez-Torres M., Acosta-Torres L.S., Diaz-Torres L.A., Grillo R., Swamy M.K., Sharma S. (2018). Nano based drug delivery systems: Recent developments and future prospects. J. Nanobiotechnol..

[34] Rizvi S.A.A., Saleh A.M. (2018). Applications of nanoparticle systems in drug delivery technology. Saudi Pharm. J..

[35] Salouti M., Ahangari A. (2014). Nanoparticle based Drug Delivery Systems for Treatment of Infectious Diseases. Appl. Nanotechnol. Drug Deliv..

[36] Salehi R., Rasouli S., Hamishehkar H. (2015). Smart thermo/pH responsive magnetic nanogels for the simultaneous delivery of doxorubicin and methotrexate. Int. J. Pharm..

[37] Helmlinger G., Yuan F., Dellian M., Jain R.K. (1997). Interstitial pH and pO_2_ gradients in solid tumors in vivo: High-resolution measurements reveal a lack of correlation. Nat. Med..

[38] Lee E.S., Na K., Bae Y.H. (2005). Doxorubicin loaded pH-sensitive polymeric micelles for reversal of resistant MCF-7 tumor. J. Control. Release.

[39] Liu Y., Cao X., Luo M., Le Z., Xu W. (2009). Self-assembled micellar nanoparticles of a novel star copolymer for thermo and pH dual-responsive drug release. J. Colloid Interface Sci..

[40] Matsubara T., Diresta G.R., Kakunaga S., Li D., Healey J.H. (2013). Additive Influence of Extracellular pH, Oxygen Tension, and Pressure on Invasiveness and Survival of Human Osteosarcoma Cells. Front. Oncol..

[41] Sun J., Gui R., Jin H., Li N., Wang X. (2016). CuS nanocrystal@microgel nanocomposites for light-regulated release of dual-drugs and chemo-photothermal synergistic therapy in vitro. RSC Adv..

[42] El-Sherbiny I.M., Smyth H.D.C. (2011). Smart Magnetically Responsive Hydrogel Nanoparticles Prepared by a Novel Aerosol-Assisted Method for Biomedical and Drug Delivery Applications. J. Nanomater..

[43] Guo S., Qiao Y., Wang W., He H., Deng L., Xing J., Xu J., Liang X.J., Dong A. (2010). Poly(ε-caprolactone)-graft-poly(2-(N, N-dimethylamino) ethyl methacrylate) nanoparticles: PH dependent thermo-sensitive multifunctional carriers for gene and drug delivery. J. Mater. Chem..

[44] Xiong W., Wang W., Wang Y., Zhao Y., Chen H., Xu H., Yang X. (2011). Dual temperature/pH-sensitive drug delivery of poly(*N*-isopropylacrylamide-co-acrylic acid) nanogels conjugated with doxorubicin for potential application in tumor hyperthermia therapy. Colloids Surf. B Biointerfaces.

[45] Zheng Y., Wang L., Lu L., Wang Q., Benicewicz B.C. (2017). pH and Thermal Dual-Responsive Nanoparticles for Controlled Drug Delivery with High Loading Content. ACS Omega.

[46] Kumar R., Shin W.S., Sunwoo K., Kim W.Y., Bhuniya S., Kim J. S. (2015). Small conjugate-based theranostic agents: An encouraging approach for cancer therapy. Chem. Soc. Rev..

[47] Xie J., Zhang Y., Yan C., Song L., Wen S., Zang F., Chen G., Ding Q., Yan C., Gu N. (2014). High-performance PEGylated Mn-Zn ferrite nanocrystals as a passive-targeted agent for magnetically induced cancer theranostics. Biomaterials.

[48] Kazunori K., Glenn S.K., Masayuki Y., Teruo O., Yasuhisa S. (1993). Block copolymer micelles as vehicles for drug delivery. J. Control. Release.

[49] Kwon G.S., Kataoka K. (1995). Block copolymer micelles as long-circulating drug vehicles. Adv. Drug Deliv. Rev..

[50] Kataoka K., Harada A., Nagasaki Y. (2001). Block copolymer micelles for drug delivery: Design, characterization and biological significance. Adv. Drug Deliv. Rev..

[51] Cabral H., Miyata K., Osada K., Kataoka K. (2018). Block Copolymer Micelles in Nanomedicine Applications. Chem. Rev..

[52] Hartshorn C.M., Bradbury M.S., Lanza G.M., Nel A.E., Rao J., Wang A.Z., Wiesner U.B., Yang L., Grodzinski P. (2018). Nanotechnology Strategies to Advance Outcomes in Clinical Cancer Care. ACS Nano.

[53] Xin Y., Yin M., Zhao L., Meng F., Luo L. (2017). Recent progress on nanoparticle-based drug delivery systems for cancer therapy. Cancer Biol. Med..

[54] Parvanian S., Mostafavi S.M., Aghashiri M. (2017). Multifunctional nanoparticle developments in cancer diagnosis and treatment. Sens. Bio-Sens. Res..

[55] Park W., Heo Y.J., Han D.K. (2018). New opportunities for nanoparticles in cancer immunotherapy. Biomater. Res..

[56] Buabeid M.A., Arafa E.S.A., Murtaza G. (2020). Emerging Prospects for Nanoparticle-Enabled Cancer Immunotherapy. J. Immunol. Res..

[57] Rocha M., Chaves N., Bào S. (2017). Nanobiotechnology for Breast Cancer Treatment. Breast Cancer Biol. Med..

[58] Rink J.S., Plebanek M.P., Tripathy S., Thaxton C.S. (2013). Update on current and potential nanoparticle cancer therapies. Curr. Opin. Oncol..

[59] Abadeer N.S., Murphy C.J. (2016). Recent Progress in Cancer Thermal Therapy Using Gold Nanoparticles. J. Phys. Chem. C.

[60] Amreddy N., Babu A., Muralidharan R., Munshi A., Ramesh R. (2017). Polymeric Nanoparticle-Mediated Gene Delivery for Lung Cancer Treatment. Top. Curr. Chem..

[61] Thorn C.F., Oshiro C., Marsh S., Hernandez-Boussard T., McLeod H., Klein T.E., Altman R.B. (2011). Doxorubicin pathways: Pharmacodynamics and adverse effects. Pharmacogenet. Genom..

[62] Tacar O., Sriamornsak P., Dass C.R. (2013). Doxorubicin: An update on anticancer molecular action, toxicity and novel drug delivery systems. J. Pharm. Pharmacol..

[63] Epstein J.B., Thariat J., Bensadoun R.J., Barasch A., Murphy B.A., Kolnick L., Popplewell L., Maghami E. (2012). Oral complications of cancer and cancer therapy: From cancer treatment to survivorship. CA Cancer J. Clin..

[64] Malhotra V., Perry M.C. (2003). Classical chemotherapy: Mechanisms, toxicities and the therapeutic window. Cancer Biol. Ther..

[65] de Golian E., Kwong B.Y., Swetter S.M., Pugliese S.B. (2016). Cutaneous Complications of Targeted Melanoma Therapy. Curr. Treat. Options Oncol..

[66] Goldinger S.M., Stieger P., Meier B., Micaletto S., Contassot E., French L.E., Dummer R. (2016). Cytotoxic cutaneous adverse drug reactions during anti-PD-1 therapy. Clin. Cancer Res..

[67] Wang D.Y., Okoye G.D., Neilan T.G., Johnson D.B., Moslehi J.J. (2017). Cardiovascular Toxicities Associated with Cancer Immunotherapies. Curr. Cardiol. Rep..

[68] Gallagher J.G. (1999). Diarrhea. Supportive Care in Cancer: A Handbook for Oncologists.

[69] Benson A.B., Ajani J.A., Catalano R.B., Engelking C., Kornblau S.M., Martenson J.A., McCallum R., Mitchell E.P., O’Dorisio T.M., Vokes E.E. (2004). Recommended guidelines for the treatment of cancer treatment-induced diarrhea. J. Clin. Oncol..

[70] Karakunnel J., Modi A.A. (2008). Diarrhea and constipation. Cancer Supportive Care: Advances in Therapeutic Strategies.

[71] Batchelor D. (2001). Hair and cancer chemotherapy: Consequences and nursing care—A literature study. Eur. J. Cancer Care.

[72] Trusson D., Pilnick A. (2017). The role of hair loss in cancer identity. Cancer Nurs..

[73] Botchkarev V.A. (2003). Molecular mechanisms of chemotherapy-induced hair loss. J. Investig. Dermatol. Symp. Proc..

[74] Richardson J.L., Marks G., Levine A. (1988). The influence of symptoms of disease and side effects of treatment on compliance with cancer therapy. J. Clin. Oncol..

[75] Minami M., Matsumoto S., Horiuchi H. (2010). Cardiovascular side-effects of modern cancer therapy. Circ. J..

[76] Nichols J.W., Bae Y.H. (2014). EPR: Evidence and fallacy. J. Control. Release.

[77] Danhier F. (2016). To exploit the tumor microenvironment: Since the EPR effect fails in the clinic, what is the future of nanomedicine?. J. Control. Release.

[78] Chadha N., Chaturvedi S., Lal S., Mishra A.K., Pulicharla R., Cledon M., Brar S.K., Surampalli R.Y. (2016). Engineered nanoparticles associated metabolomics. J. Hazard. Toxic Radioact. Waste.

[79] Pucci C., Martinelli C., Ciofani G. (2019). Innovative approaches for cancer treatment: Current perspectives and new challenges. Ecancermedicalscience.

[80] Greten T.F., Wang X.W., Korangy F. (2015). Current concepts of immune based treatments for patients with HCC: From basic science to novel treatment approaches. Gut.

[81] Taieb J., Barbare J.C., Rougier P. (2006). Medical treatments for hepatocellular carcinoma (HCC): What’s next?. Ann. Oncol..

[82] Simel D.L., Goldman L., Schafer A.I. (2019). Approach to the Patient: Hystory and Physical Esamination. Goldman-Cecil Medicine.

[83] Franks T. What Is Standard Therapy in Cancer Treatment?. https://www.td2inc.com/news/what-is-standard-therapy-in-cancer-treatment#:~:text=Standard.

[84] National Cancer Institute Types of Cancer Treatment. https://www.cancer.gov/about-cancer/treatment/types.

[85] Medline Plus Cancer Treatments. https://medlineplus.gov/ency/patientinstructions/000901.htm.

[86] Ventola C.L. (2017). Progress in nanomedicine: Approved and investigational nanodrugs. Pharm. Ther..

[87] Martins J.P., das Neves J., de la Fuente M., Celia C., Florindo H., Günday-Türeli N., Popat A., Santos J.L., Sousa F., Schmid R. (2020). The solid progress of nanomedicine. Drug Deliv. Transl. Res..

[88] Hare J.I., Lammers T., Ashford M.B., Puri S., Storm G., Barry S.T. (2017). Challenges and strategies in anti-cancer nanomedicine development: An industry perspective. Adv. Drug Deliv. Rev..

[89] Wang R., Billone P.S., Mullett W.M. (2013). Nanomedicine in action: An overview of cancer nanomedicine on the market and in clinical trials. J. Nanomater..

[90] Greish K., Mathur A., Bakhiet M., Taurin S. (2018). Nanomedicine: Is it lost in translation?. Ther. Deliv..

[91] Sarmento B. (2019). Have nanomedicines progressed as much as we’d hoped for in drug discovery and development?. Expert Opin. Drug Discov..

[92] Wicki A., Witzigmann D., Balasubramanian V., Huwyler J. (2015). Nanomedicine in cancer therapy: Challenges, opportunities, and clinical applications. J. Control. Release.

[93] Krishnamachari Y., Geary S.M., Lemke C.D., Salem A.K. (2011). Nanoparticle delivery systems in cancer vaccines. Pharm. Res..

[94] Shafei A., El-Bakly W., Sobhy A., Wagdy O., Reda A., Aboelenin O., Marzouk A., El Habak K., Mostafa R., Ali M.A. (2017). A review on the efficacy and toxicity of different doxorubicin nanoparticles for targeted therapy in metastatic breast cancer. Biomed. Pharmacother..

[95] Resnik D.B., Tinkle S.S. (2007). Ethical issues in clinical trials involving nanomedicine. Contemp. Clin. Trials.

[96] Vert M., Doi Y., Hellwich K.H., Hess M., Hodge P., Kubisa P., Rinaudo M., Schué F. (2012). Terminology for biorelated polymers and applications (IUPAC recommendations 2012). Pure Appl. Chem..

[97] Roduner E. (2006). Size matters: Why nanomaterials are different. Chem. Soc. Rev..

[98] Hernando A., Crespo P., García M.A. (2005). Metallic magnetic nanoparticles. Sci. World J..

[99] Sun Y., Gray S.K., Peng S. (2011). Surface chemistry: A non-negligible parameter in determining optical properties of small colloidal metal nanoparticles. Phys. Chem. Chem. Phys..

[100] Bastus N.G., Casals E., Ojea I., Varon M., Puntes V., Abbass A.H. (2012). The Reactivity of Colloidal Inorganic Nanoparticles. The Delivery of Nanoparticles.

[101] Alberts B. (2013). Impact factor distortions. Science.

[102] Vigers G.P., Crowther R.A., Pearse B.M. (1986). Location of the 100 kd-50 kd accessory proteins in clathrin coats. EMBO J..

[103] Brannon-Peppas L. (1995). Recent advances on the use of biodegradable microparticles and nanoparticles in controlled drug delivery. Int. J. Pharm..

[104] Rao K.V.R., Buri P. (1989). A novel in situ method to test polymers and coated microparticles for bioadhesion. Int. J. Pharm..

[105] Melchiorre F., Patella F., Pescatori L., Pesapane F., Fumarola E., Biondetti P., Brambillasca P., Monaco C., Ierardi A.M., Franceschelli G. (2018). DEB-TACE: A standard review. Futur. Oncol..

[106] Fuchs K., Duran R., Denys A., Bize P.E., Borchard G., Jordan O. (2017). Drug-eluting embolic microspheres for local drug delivery—State of the art. J. Control. Release.

[107] Lee B.K., Yun Y.H., Park K. (2015). Smart nanoparticles for drug delivery: Boundaries and opportunities. Chem. Eng. Sci..

[108] Choi H.S., Liu W., Liu F., Nasr K., Misra P., Bawendi M.G., Frangioni J.V. (2010). Design considerations for tumour-targeted nanoparticles. Nat. Nanotechnol..

[109] Du B., Yu M., Zheng J. (2018). Transport and interactions of nanoparticles in the kidneys. Nat. Rev. Mater..

[110] Hainfeld J.F., Slatkin D.N., Smilowitz H.M. (2004). The use of gold nanoparticles to enhance radiotherapy in mice. Phys. Med. Biol..

[111] Lankveld D.P.K., Oomen A.G., Krystek P., Neigh A., Troost de Jong A., Noorlander C.W., Van Eijkeren J.C.H., Geertsma R.E., De Jong W.H. (2010). The kinetics of the tissue distribution of silver nanoparticles of different sizes. Biomaterials.

[112] Tiwari G., Tiwari R., Bannerjee S., Bhati L., Pandey S., Pandey P., Sriwastawa B. (2012). Drug delivery systems: An updated review. Int. J. Pharm. Investig..

[113] Kreuter J., Speiser P.P. (1976). In vitro studies of poly(methyl methacrylate) adjuvants. J. Pharm. Sci..

[114] Choi H.S., Ashitate Y., Lee J.H., Kim S.H., Matsui A., Insin N., Bawendi M.G., Semmler-Behnke M., Frangioni J.V., Tsuda A. (2010). Rapid translocation of nanoparticles from the lung airspaces to the body. Nat. Biotechnol..

[115] Cosson P., Amherdt M., Rothman J.E., Orci L. (2002). A resident Golgi protein is excluded from peri-Golgi vesicles in NRK cells. Proc. Natl. Acad. Sci. USA.

[116] Takamori S., Holt M., Stenius K., Lemke E.A., Grønborg M., Riedel D., Urlaub H., Schenck S., Brügger B., Ringler P. (2006). Molecular Anatomy of a Trafficking Organelle. Cell.

[117] Gaumet M., Vargas A., Gurny R., Delie F. (2008). Nanoparticles for drug delivery: The need for precision in reporting particle size parameters. Eur. J. Pharm. Biopharm..

[118] Oberdorster G., Ferin J., Lehnert B.E. (1994). Correlation between particle size, in vivo particle persistence, and lung injury. Environ. Health Perspect..

[119] Oberdörster G., Sharp Z., Atudorei V., Elder A., Gelein R., Lunts A. (2002). Extrapulmonary translocation of ultrafine carbon particles following whole-body inhalation exposure of rats. J. Toxicol. Environ. Health Part A.

[120] Kreyling W.G., Semmler M., Erbe F., Mayer P., Takenaka S., Schulz H., Oberdörster G., Ziesenis A. (2002). Translocation of ultrafine insoluble iridium particles from lung epithelium to extrapulmonary organs is size dependent but very low. J. Toxicol. Environ. Health Part A.

[121] Pant K., Pufe J., Zarschler K., Bergmann R., Steinbach J., Reimann S., Haag R., Pietzsch J., Stephan H. (2017). Surface charge and particle size determine the metabolic fate of dendritic polyglycerols. Nanoscale.

[122] Blanco E., Shen H., Ferrari M. (2015). Principles of nanoparticle design for overcoming biological barriers to drug delivery. Nat. Biotechnol..

[123] Wilhelm S., Tavares A.J., Dai Q., Ohta S., Audet J., Dvorak H.F., Chan W.C.W. (2016). Analysis of nanoparticle delivery to tumours. Nat. Rev. Mater..

[124] Brannon-Peppas L., Blanchette J.O. (2004). Nanoparticle and targeted systems for cancer therapy. Adv. Drug Deliv. Rev..

[125] Storm G., Belliot S., Daemenb T., Lasic D.D. (1995). Surface modification of nanoparticles to oppose uptake by the mononuclear phagocyte system. Adv. Drug Deliv. Rev..

[126] Klibanovl A.L., Maruyamal K., Torchilin V.P., Huangl L. (1990). Amphipathic polyethyleneglycols effectively prolong the circulation time of liposomes. FEBS Lett..

[127] Capolla S., Garrovo C., Zorzet S., Lorenzon A., Rampazzo E., Spretz R., Pozzato G., Núñez L., Tripodo C., Macor P. (2015). Targeted tumor imaging of anti-CD20-polymeric nanoparticles developed for the diagnosis of B-cell malignancies. Int. J. Nanomed..

[128] Kirpotin D.B., Drummond D.C., Shao Y., Shalaby M.R., Hong K., Nielsen U.B., Marks J.D., Benz C.C., Park J.W. (2006). Antibody targeting of long-circulating lipidic nanoparticles does not increase tumor localization but does increase internalization in animal models. Cancer Res..

[129] Bartlett D.W., Su H., Hildebrandt I.J., Weber W.A., Davis M.E. (2007). Impact of tumor-specific targeting on the biodistribution and efficacy of siRNA nanoparticles measured by multimodality in vivo imaging. Proc. Natl. Acad. Sci. USA.

[130] Kunjachan S., Pola R., Gremse F., Theek B., Ehling J., Moeckel D., Hermanns-Sachweh B., Pechar M., Ulbrich K., Hennink W.E. (2014). Passive versus active tumor targeting using RGD- and NGR-modified polymeric nanomedicines. Nano Lett..

[131] Yang J., Zhu Y., Wang F., Deng L., Xu X., Cui W. (2020). Microfluidic liposomes-anchored microgels as extended delivery platform for treatment of osteoarthritis. Chem. Eng. J..

[132] Silva A.C., González-Mira E., Lobo J.M., Amaral M.H. (2013). Current Progresses on Nanodelivery Systems for the Treatment of Neuropsychiatric Diseases: Alzheimer’s and Schizophrenia. Curr. Pharm. Des..

[133] Tao J., Xu X., Wang S., Kang T., Guo C., Liu X., Cheng H., Liu Y., Jiang X., Mao J. (2019). Polydiacetylene-Nanoparticle-Functionalized Microgels for Topical Bacterial Infection Treatment. ACS Macro Lett..

[134] Schwendener R.A. (2014). Liposomes as vaccine delivery systems: A review of the recent advances. Ther. Adv. Vaccines.

[135] Polack F.P., Thomas S.J., Kitchin N., Absalon J., Gurtman A., Lockhart S., Perez J.L., Pérez Marc G., Moreira E.D., Zerbini C. (2020). Safety and Efficacy of the BNT162b2 mRNA COVID-19 Vaccine. N. Engl. J. Med..

[136] Dušek K., Dušková-Smrčková M. (2020). Volume phase transition in gels: Its discovery and development. Gels.

[137] Kawaguchi H. (2020). On Going to a New Era of Microgel Exhibiting Volume Phase Transition. Gels.

[138] Constantin M., Cristea M., Ascenzi P., Fundueanu G. (2011). Lower critical solution temperature versus volume phase transition temperature in thermoresponsive drug delivery systems. Express Polym. Lett..

[139] Ramos J., Imaz A., Forcada J. (2012). Temperature-sensitive nanogels: Poly(*N*-vinylcaprolactam) versus poly(*N*-isopropylacrylamide). Polym. Chem..

[140] Drozdov A.D., Christiansen J.D. (2021). Modulation of the volume phase transition temperature of thermo-responsive gels. J. Mech. Behav. Biomed. Mater..

[141] Khan A. (2008). Preparation and characterization of magnetic nanoparticles embedded in microgels. Mater. Lett..

[142] Sanoj Rejinold N., Thomas R.G., Muthiah M., Chennazhi K.P., Manzoor K., Park I.K., Jeong Y.Y., Jayakumar R. (2015). Anti-cancer, pharmacokinetics and tumor localization studies of pH-, RF- and thermo-responsive nanoparticles. Int. J. Biol. Macromol..

[143] Šolc K., Dušek K., Koningsveld R., Berghmans H. (1995). “Zero” and “Off-Zero” Critical Concentrations in Solutions of Polydisperse Polymers with Very High Molar Masses. Collect. Czechoslov. Chem. Commun..

[144] Moerkerke R., Meeussen F., Koningsveld R., Berghmans H., Mondelaers W., Schacht E., Dusek K., Solc K. (1998). Phase transitions in swollen networks. 3. Swelling behavior of radiation cross-linked poly(vinyl methyl ether) in water. Macromolecules.

[145] Meeussen F., Nies E., Berghmans H., Verbrugghe S., Goethals E., Du Prez F. (2000). Phase behaviour of poly(N-vinyl caprolactam) in water. Polymer.

[146] Ainara I., Jacqueline F. (2010). *N*-vinylcaprolactam-based microgels for biomedical applications. J. Polym. Sci. Part A Polym. Chem..

[147] Corezzi S., Comez L., Zanatta M. (2018). A simple analysis of Brillouin spectra from opaque liquids and its application to aqueous suspensions of poly-*N*-isopropylacrylamide microgel particles. J. Mol. Liq..

[148] Stetefeld J., McKenna S.A., Patel T.R. (2016). Dynamic light scattering: A practical guide and applications in biomedical sciences. Biophys. Rev..

[149] Moreno A.J., Lo Verso F. (2018). Computational investigation of microgels: Synthesis and effect of the microstructure on the deswelling behavior. Soft Matter.

[150] Rampino A., Borgogna M., Blasi P., Bellich B., Cesàro A. (2013). Chitosan nanoparticles: Preparation, size evolution and stability. Int. J. Pharm..

[151] Nizri G., Magdassi S., Schmidt J., Cohen Y., Talmon Y. (2004). Microstructural characterization of micro- and nanoparticles formed by polymer-surfactant interactions. Langmuir.

[152] Holderer O., Maccarrone S., Pasini S., Appavou M.S., Gelissen A. (2021). Raspberry structures in microgel–silica nanoparticle composite systems. Results Phys..

[153] Ninarello A., Crassous J.J., Paloli D., Camerin F., Gnan N., Rovigatti L., Schurtenberger P., Zaccarelli E. (2019). Modeling Microgels with a Controlled Structure across the Volume Phase Transition. Macromolecules.

[154] Svergun D.I., Richard S., Koch M.H.J., Sayers Z., Kuprin S., Zaccai G. (1998). Protein hydration in solution: Experimental observation by x-ray and neutron scattering. Proc. Natl. Acad. Sci. USA.

[155] Brugnoni M., Nickel A.C., Kröger L.C., Scotti A., Pich A., Leonhard K., Richtering W. (2019). Synthesis and structure of deuterated ultra-low cross-linked poly( N -isopropylacrylamide) microgels. Polym. Chem..

[156] Cors M., Wiehemeier L., Hertle Y., Feoktystov A., Cousin F., Hellweg T., Oberdisse J. (2018). Determination of Internal Density Profiles of Smart Acrylamide-Based Microgels by Small-Angle Neutron Scattering: A Multishell Reverse Monte Carlo Approach. Langmuir.

[157] Jonassen H., Kjøniksen A.L., Hiorth M. (2012). Effects of ionic strength on the size and compactness of chitosan nanoparticles. Colloid Polym. Sci..

[158] Calvo P., Remuñán-López C., Vila-Jato J.L., Alonso M.J. (1997). Novel hydrophilic chitosan-polyethylene oxide nanoparticles as protein carriers. J. Appl. Polym. Sci..

[159] Grobelny S., Hofmann C.H., Erlkamp M., Plamper F.A., Richtering W., Winter R. (2013). Conformational changes upon high pressure induced hydration of poly(*N*-isopropylacrylamide) microgels. Soft Matter.

[160] Suzuki D., Nagase Y., Kureha T., Sato T. (2014). Internal structures of thermosensitive hybrid microgels investigated by means of small-angle X-ray scattering. J. Phys. Chem. B.

[161] Kikhney A.G., Svergun D.I. (2015). A practical guide to small angle X-ray scattering (SAXS) of flexible and intrinsically disordered proteins. FEBS Lett..

[162] Zhuo S., Zhang F., Yu J., Zhang X., Yang G. (2020). pH-Sensitive Biomaterials for Drug Delivery. Molecules.

[163] Jiang X., Lu G., Feng C., Li Y., Huang X. (2013). Poly(acrylic acid)-graft-poly(*N*-vinylcaprolactam): A novel pH and thermo dual-stimuli responsive system. Polym. Chem..

[164] Yang G., Wang X., Fu S., Tang R., Wang J. (2017). pH-triggered chitosan nanogels via an ortho ester-based linkage for efficient chemotherapy. Acta Biomater..

[165] Dirksen M., Dargel C., Meier L., Brändel T., Hellweg T. (2020). Smart microgels as drug delivery vehicles for the natural drug aescin: Uptake, release and interactions. Colloid Polym. Sci..

[166] Dalmont H., Pinprayoon O., Saunders B.R. (2008). Study of pH-responsive microgels containing methacrylic acid: Effects of particle composition and added calcium. Langmuir.

[167] Vihola H., Laukkanen A., Valtola L., Tenhu H., Hirvonen J. (2005). Cytotoxicity of thermosensitive polymers poly(*N*-isopropylacrylamide), poly(*N*-vinylcaprolactam) and amphiphilically modified poly(*N*-vinylcaprolactam). Biomaterials.

[168] Rao K., Rao K., Ha C.-S. (2016). Stimuli Responsive Poly(Vinyl Caprolactam) Gels for Biomedical Applications. Gels.

[169] Karg M., Pich A., Hellweg T., Hoare T., Lyon L.A., Crassous J.J., Suzuki D., Gumerov R.A., Schneider S., Potemkin I.I. (2019). Nanogels and Microgels: From Model Colloids to Applications, Recent Developments, and Future Trends. Langmuir.

[170] Bao H., Li L., Leong W.C., Gan L.H. (2010). Thermo-responsive association of chitosan-graft-poly(N -isopropylacrylamide) in aqueous solutions. J. Phys. Chem. B.

[171] Jaiswal M.K., Mehta S., Banerjee R., Bahadur D. (2012). A comparative study on thermoresponsive magnetic nanohydrogels: Role of surface-engineered magnetic nanoparticles. Colloid Polym. Sci..

[172] Sreekumar S., Goycoolea F.M., Moerschbacher B.M., Rivera-Rodriguez G.R. (2018). Parameters influencing the size of chitosan-TPP nano- and microparticles. Sci. Rep..

[173] Islam N., Wang H., Maqbool F., Ferro V. (2019). In vitro enzymatic digestibility of glutaraldehyde-crosslinked chitosan nanoparticles in lysozyme solution and their applicability in pulmonary drug delivery. Molecules.

[174] Islam N., Dmour I., Taha M.O. (2019). Degradability of chitosan micro/nanoparticles for pulmonary drug delivery. Heliyon.

[175] Desai K.G.H. (2016). Chitosan nanoparticles prepared by ionotropic gelation: An overview of recent advances. Crit. Rev. Ther. Drug Carr. Syst..

[176] Ajun W., Yan S., Li G., Huili L. (2009). Preparation of aspirin and probucol in combination loaded chitosan nanoparticles and in vitro release study. Carbohydr. Polym..

[177] Pan Y., Li Y., Zhao H., Zheng J., Xu H., Wei G., Hao J., Cui F. (2002). Bioadhesive polysaccharide in protein delivery system: Chitosan nanoparticles improve the intestinal absorption of insulin in vivo. Int. J. Pharm..

[178] Huang Y., Lapitsky Y. (2011). Monovalent salt enhances colloidal stability during the formation of chitosan/tripolyphosphate microgels. Langmuir.

[179] Jonassen H., Kjøniksen A.L., Hiorth M. (2012). Stability of chitosan nanoparticles cross-linked with tripolyphosphate. Biomacromolecules.

[180] Sacco P., Cok M., Asaro F., Paoletti S., Donati I. (2018). The role played by the molecular weight and acetylation degree in modulating the stiffness and elasticity of chitosan gels. Carbohydr. Polym..

[181] Calvo P., Remuñan-López C., Vila-Jato J.L., Alonso M.J. (1997). Chitosan and Chitosan/Ethylene Oxide-Propylene oxide Block Copolymer Nanoparticles as Novel Carriers for Proteins and Vaccines. Pharm. Res..

[182] Zhang H., Oh M., Allen C., Kumacheva E. (2004). Monodisperse chitosan nanoparticles for mucosal drug delivery. Biomacromolecules.

[183] Fan W., Yan W., Xu Z., Ni H. (2012). Formation mechanism of monodisperse, low molecular weight chitosan nanoparticles by ionic gelation technique. Colloids Surf. B Biointerfaces.

[184] Tsai M.L., Bai S.W., Chen R.H. (2008). Cavitation effects versus stretch effects resulted in different size and polydispersity of ionotropic gelation chitosan-sodium tripolyphosphate nanoparticle. Carbohydr. Polym..

[185] Horinek D., Kreysa G., Ota K., Savinell R.F. (2014). DLVO Theory. Encyclopedia of Applied Electrochemistry.

[186] Wang M., Qiang J., Fang Y.U., Hu D., Cui Y., Fu X. (1999). Preparation and Properties of Chitosan-Poly (*N*-isopropylacrylamide) Semi-IPN Hydrogels. J. Polym. Sci. Part A Polym. Chem..

[187] Berger J., Reist M., Mayer J.M., Felt O., Peppas N.A., Gurny R. (2004). Structure and interactions in covalently and ionically crosslinked chitosan hydrogels for biomedical applications. Eur. J. Pharm. Biopharm..

[188] Bellich B., D’Agostino I., Semeraro S., Gamini A., Cesàro A. (2016). “The good, the Bad and the Ugly” of Chitosans.

[189] Korsmeyer R.W., Gurny R., Doelker E., Buri P., Peppas N.A. (1983). Mechanisms of solute release from porous hydrophilic polymers. Int. J. Pharm..

[190] Higuchi T. (1963). Mechanism of sustained-action medication. Theoretical analysis of rate of release of solid drugs dispersed in solid matrices. J. Pharm. Sci..

[191] Mohammed M.N., Bin Yusoh K., Shariffuddin J.H.B.H. (2018). Poly(N-vinyl caprolactam) thermoresponsive polymer in novel drug delivery systems: A review. Mater. Express.

[192] Rejinold N.S., Chennazhi K.P., Nair S.V., Tamura H., Jayakumar R. (2011). Biodegradable and thermo-sensitive chitosan-g-poly(*N*-vinylcaprolactam) nanoparticles as a 5-fluorouracil carrier. Carbohydr. Polym..

[193] Prabaharan M., Mano J.F. (2006). Stimuli-responsive hydrogels based on polysaccharides incorporated with thermo-responsive polymers as novel biomaterials. Macromol. Biosci..

[194] Prabaharan M., Jayakumar R., Jayakumar R., Prabaharan M., Muzzarelli A.A.R. (2011). Polymeric Bionanocomposites as Promising Materials for Controlled Drug Delivery. Chitosan for BIOMATERIALS II.

[195] Chen J.P., Cheng T.H. (2006). Thermo-responsive chitosan-graft-poly(*N*-isopropylacrylamide) injectable hydrogel for cultivation of chondrocytes and meniscus cells. Macromol. Biosci..

[196] Malhotra M., Lane C., Tomaro-Duchesneau C., Saha S., Prakash S. (2011). A novel method for synthesizing PEGylated chitosan nanoparticles: Strategy, preparation, and in vitro analysis. Int. J. Nanomed..

[197] Hirai A., Odani H., Nakajima A. (1991). Determination of degree of deacetylation of chitosan by 1H NMR spectroscopy. Polym. Bull..

[198] Xu Y., Du Y. (2003). Effect of molecular structure of chitosan on protein delivery properties of chitosan nanoparticles. Int. J. Pharm..

[199] Ugelstad J. (1984). Monodisperse Polymer Particles and Dispersions Thereof. U.S. Patent.

[200] Kozanoǧlu S., Özdemir T., Usanmaz A. (2011). Polymerization of *N*-vinylcaprolactam and characterization of poly(*N*-vinylcaprolactam). J. Macromol. Sci. Part A Pure Appl. Chem..

[201] Kirsh Y.E. (1998). Water Soluble Poly-N-Vinylamides: Synthesis and Physicochemical Properties.

[202] Marsili L., Bo M.D., Eisele G., Donati I., Berti F., Toffoli G. (2021). Characterization of thermoresponsive poly-N- Vinylcaprolactam polymers for biological applications. Polymers.

[203] Shao L., Hu M., Chen L., Xu L., Bi Y. (2012). RAFT polymerization of *N*-vinylcaprolactam and effects of the end group on the thermal response of poly(*N*-vinylcaprolactam). React. Funct. Polym..

[204] Feil H., Bae Y.H., Feijen J., Kim S.W. (1993). Effect of comonomer hydrophilicity and ionization on the lower critical solution temperature of *N*-isopropylacrylamide copolymers. Macromolecules.

[205] Niu S., Williams G.R., Wu J., Wu J., Zhang X., Chen X., Li S., Jiao J., Zhu L.M. (2019). A chitosan-based cascade-responsive drug delivery system for triple-negative breast cancer therapy. J. Nanobiotechnol..

[206] Sahebi H., Pourmortazavi S.M., Zandavar H., Mirsadeghi S. (2019). Chitosan grafted onto Fe3O4@poly(: *N*-vinylcaprolactam) as a new sorbent for detecting Imatinib mesylate in biosamples using UPLC-MS/MS. Analyst.

[207] Banihashem S., Nikpour Nezhati M., Panahi H.A., Shakeri-Zadeh A. (2020). Synthesis of novel chitosan-g-PNVCL nanofibers coated with gold-gold sulfide nanoparticles for controlled release of cisplatin and treatment of MCF-7 breast cancer. Int. J. Polym. Mater. Polym. Biomater..

[208] Banihashem S., Nezhati M.N., Panahia H.A. (2020). Synthesis of chitosan-grafted-poly(*N*-vinylcaprolactam) coated on the thiolated gold nanoparticles surface for controlled release of cisplatin. Carbohydr. Polym..

[209] Janes K.A., Fresneau M.P., Marazuela A., Fabra A., Alonso M.J. (2001). Chitosan nanoparticles as delivery systems for doxorubicin. J. Control. Release.

[210] Bahmani E., Koushkbaghi S., Darabi M., ZabihiSahebi A., Askari A., Irani M. (2019). Fabrication of novel chitosan-g-PNVCL/ZIF-8 composite nanofibers for adsorption of Cr(VI), As(V) and phenol in a single and ternary systems. Carbohydr. Polym..

[211] Hermosillo-Ochoa E., Picos-Corrales L.A., Licea-Claverie A. (2021). Eco-friendly flocculants from chitosan grafted with PNVCL and PAAc: Hybrid materials with enhanced removal properties for water remediation. Sep. Purif. Technol..

[212] Menozzi M., Valentini L., Vannini E., Arcamone F. (1984). Self-association of doxorubicin and related compounds in aqueous solution. J. Pharm. Sci..

[213] Martin H. (1985). Tetracyclines and daunorubicin. Met. Ions Biol. Syst..

[214] Daugherty J.P., Hixon S.C., Yielding K.L. (1979). Direct in vitro photoaffinity labeling of DNA with daunorubicin, adriamycin, and rubidazone. BBA Sect. Nucleic Acids Protein Synth..

[215] Thoma K., Stritmatter T., Steinbach D. (1980). Untersuchungen zur photoinstabilitat von antibiotika. Acta Pharm. Technol..

[216] Tavoloni N., Guarino A.M., Berk P.D. (1980). Photolytic degradation of adriamycin. J. Pharm. Pharmacol..

[217] Arcamone F. (1978). Daunomycin and related antibiotics. Top. Antibiot. Chem..

[218] Beijnen J.H., van der Houwen O.A.G.J., Underberg W.J.M. (1986). Aspects of the degradation kinetics of doxorubicin in aqueous solution. Int. J. Pharm..

[219] Beijnen J.H., Wiese G., Underberg W.J.M. (1985). Aspects of the chemical stability of doxorubicin and seven other anthracydines in acidic solution. Pharm. Weekbl. Sci. Ed..

[220] Sanyakamdhorn S., Agudelo D., Tajmir-Riahi H.A. (2013). Encapsulation of antitumor drug doxorubicin and its analogue by chitosan nanoparticles. Biomacromolecules.

[221] Karnati K.R., Wang Y. (2018). Understanding the co-loading and releasing of doxorubicin and paclitaxel using chitosan functionalized single-walled carbon nanotubes by molecular dynamics simulations. Phys. Chem. Chem. Phys..

[222] Almeida A., Araújo M., Novoa-Carballal R., Andrade F., Gonçalves H., Reis R.L., Lúcio M., Schwartz S., Sarmento B. (2020). Novel amphiphilic chitosan micelles as carriers for hydrophobic anticancer drugs. Mater. Sci. Eng. C.

[223] Eliaz R.E., Szoka J. (2001). Liposome-encapsulated doxorubicin targeted to CD44: A strategy to kill CD44-overexpressing tumor cells. Cancer Res..

[224] Etrych T., Chytil P., Jelínková M., Říhová B., Ulbrich K. (2002). Synthesis of HPMA copolymers containing doxorubicin bound via a hydrazone linkage. Effect of spacer on drug release and in vitro cytotoxicity. Macromol. Biosci..

[225] Subr V., Strohalm J., Ulbrich K. (1992). Polymer containing enzymatically degradable bonds, XII. Effect of spacer structure on the rate of release of daunomycin and adriamycin from poly. J. Control. Release.

